# Progress in Soybean Genetic Transformation Over the Last Decade

**DOI:** 10.3389/fpls.2022.900318

**Published:** 2022-06-09

**Authors:** Hu Xu, Yong Guo, Lijuan Qiu, Yidong Ran

**Affiliations:** ^1^Tianjin Genovo Biotechnology Co., Ltd., Tianjin, China; ^2^Institute of Crop Sciences, Chinese Academy of Agricultural Sciences, Beijing, China

**Keywords:** soybean transformation, transformation efficiency, genotype, *Agrobacterium*, biolistic method, genome editing

## Abstract

Soybean is one of the important food, feed, and biofuel crops in the world. Soybean genome modification by genetic transformation has been carried out for trait improvement for more than 4 decades. However, compared to other major crops such as rice, soybean is still recalcitrant to genetic transformation, and transgenic soybean production has been hampered by limitations such as low transformation efficiency and genotype specificity, and prolonged and tedious protocols. The primary goal in soybean transformation over the last decade is to achieve high efficiency and genotype flexibility. Soybean transformation has been improved by modifying tissue culture conditions such as selection of explant types, adjustment of culture medium components and choice of selection reagents, as well as better understanding the transformation mechanisms of specific approaches such as Agrobacterium infection. Transgenesis-based breeding of soybean varieties with new traits is now possible by development of improved protocols. In this review, we summarize the developments in soybean genetic transformation to date, especially focusing on the progress made using *Agrobacterium*-mediated methods and biolistic methods over the past decade. We also discuss current challenges and future directions.

## Introduction

Soybean [*Glycine max* (L.) Merrill] is a legume crop belonging to the family of Leguminosae, a subfamily of Papilionoideae. Soybean is grown worldwide and is one of the most important crop plants for its high seed oil and protein content, and for its capability to fix atmospheric nitrogen by symbioses with soil-borne microorganisms. Recent studies on high-quality reference genome sequencing of a United States variety, Williams82 (Schmutz et al., 2010), a Japanese variety, Enrei (Shimomura et al., 2015), a Chinese cultivar, Zhonghuang13, and a wild soybean, W05 (Shen et al., 2018; Xie et al., 2019) have estimated that there exist a total of 46,430 protein-coding genes in soybean, 70% more than that in *Arabidopsis*. Soybean is an ancient polyploidy (palaeopolyploid) plant with a highly duplicated genome. Nearly 75% of the genes are present in multiple copies, representing a threefold redundancy due to its long evolutionary history (Schmutz et al., 2010). Some repetitive sequence families may be species-specific (Morgante et al., 1997). Several other databases have been developed, including an expressed sequence tag (EST) database, full-length cDNAs and cDNA microarrays (Stacey et al., 2004; Umezawa et al., 2008), and a haplotype map (GmHapMap) (Torkamaneh et al., 2019). These resources provide a wide range of opportunities for soybean improvement by marker-assisted breeding and with transgenic and genome editing approaches, and for understanding gene function through various forward and reverse genetic approaches. Most of these approaches are reliant on high-throughput transformation systems.

Genetic transformations allow for various genes of interest to be introduced and expressed in cells of living organisms, which can also overcome barriers of sexual incompatibility. Soybean genetic transformation was originally developed in late 1980s. The first fertile transgenic soybeans were produced by either regeneration of cotyledonary nodes infected with *Agrobacterium tumefaciens* (Hinchee et al., 1988) or by particle bombardment using meristems of immature soybean seeds (Mccabe et al., 1988). The development of soybean transgenic methods before 2013 has previously been extensively reviewed (Homrich et al., 2012; Yamada et al., 2012; Lee et al., 2013; Mariashibu et al., 2013). Soybean improvements using these transformation methods have been continued over the last 30 years. Since the first transgenic herbicide-resistant soybean product was commercialized in the mid 1990s, soybean has become one of the most important crops improved using modern biotechnology and one of the major commercially grown transgenic plants around the world. Genetically modified (GM) soybean, especially the GM Roundup Ready soybean resistant to glyphosate herbicides, has been grown in many countries including the United States, Argentina, and Brazil (Pagano and Miransari, 2016), which has made it a leading biotech crop. This soybean variety allows for growers to spray herbicides to kill any weeds in the field while not killing the soybean crop^1^. It was reported that about 105 million hectares of GM soybean was grown in 2017, and that about 272 million metric tons of seeds were produced, which accounted for 80% of all soybean production in the world (Voora et al., 2020). Genetic engineering has been conducted to enhance the protein quality of soybean by altering biosynthetic feedback pathways that increase lysine and sulfur-containing amino acids (Falco et al., 1995). Many types of GM soybeans have improved traits such as increased oleic acid content, decreased linolenic acid content, delayed flowering time, modified plant architecture and increased yield (Yamada et al., 2012). With increasing soybean demands around the world, especially from China, developing GM soybean varieties with high quality and yield is the main task for soybean researchers and breeders. Recently, genome editing (GE) technologies, especially the clustered regularly interspaced short palindromic repeats (CRISPR)/CRISPR-associated (Cas) technology, have been used for studying soybean genetics and commercial trait development (reviewed in Xu et al., 2020). However, using genome editing technologies on plants has been heavily dependent on efficient transformation systems and regeneration of plants containing edited events (Ran et al., 2017; Gao, 2021). Therefore, an efficient and genotype-flexible transformation system is key to realizing soybean improvement using these new technologies. Unfortunately, soybean remains recalcitrant to routine transformations compared to other major cultivated crops such as rice (Chen et al., 2018b). Low transformation frequency and genotype inflexibility are major hurdles that limit soybean transgenesis and breeding. In this review, we will summarize the major achievements that have been made in this field since 2013, and describe current best methods used for achieving stable and transient transformations in soybean. We also describe the remaining challenges that need to be addressed.

## Current Transformation Methods Developed for Soybean

**FIGURE 1:**
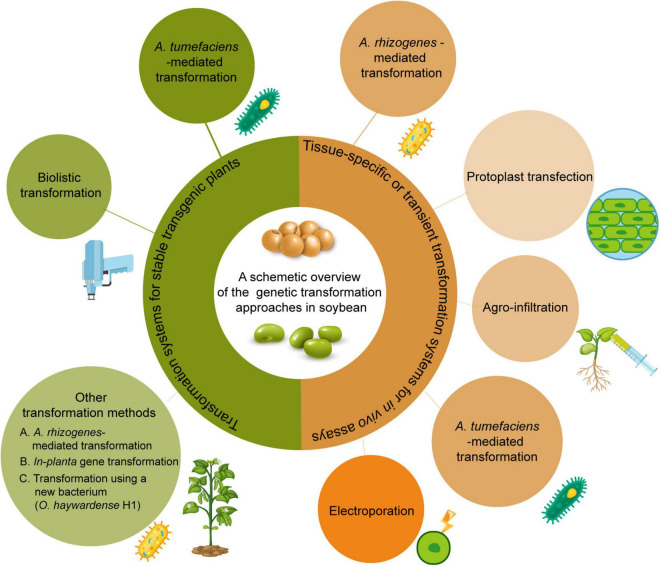
Current available genetic transformation methods for soybean. The left part with greenish color shows transformation methods to produce stable whole plants. The right part with yellowish color shows transformation methods for transient assay.

Various transformation methods have been developed for soybean. Here, we will summarize each transformation method and its ability to produce either stable transgenic plants or transient events used for soybean research (Figure 1).

### Tissue-Specific or Transient Transformation Systems for *in vivo* Assays

Transient assays are used for a variety of studies including the functional genomics of *in vivo* gene expression and subcellular gene localization, and determination of genome editing efficiency. For soybean, *Agrobacterium rhizogenes*-mediated transformation, protoplast transfection, Agro-infiltration, and electroporation have been developed. *Agrobacterium* transformation and protoplast transfection are frequently performed for transient assays.

#### *Agrobacterium rhizogenes*-Mediated Transformation

*Agrobacterium rhizogenes*-mediated transformation leads to development of a hairy-root phenotype. This method relies on co-transfer of T-DNAs from the Ri plasmid and a binary vector containing a gene of interest into the plant genome (Christey, 2001; Broothaerts et al., 2005). Large numbers of transgenic hairy-roots can be obtained in the absence of exogenous plant growth regulators (Collier et al., 2005), and each represents an independent transformation event (Kereszt et al., 2007). The relatively short timeframe (approximately 6–8 weeks) for recovering transformants is a major advantage for screening genes and promoters or expressing foreign genes in a stably transformed plant as a bioreactor (Cho et al., 2000; Bahramnejad et al., 2019). This method is also used for studying functional genomics in soybean roots. This approach has been used to characterize promoters (Hernandez-Garcia et al., 2009; Li et al., 2014), propagation of nematodes (Cho et al., 2000), symbiotic interactions (Hayashi et al., 2012), pathogenic interactions (Li et al., 2010), gene silencing by RNA interference (RNAi) (Subramanian et al., 2004), and recently for measuring genome editing activity (Du et al., 2016; Cheng et al., 2021). Recently, a reporter gene *AtMyb75*, encoding an R2R3 type MYB transcription factor, was ectopically expressed in hairy roots-mediated by *A. rhizogenes* and induced purple/red colored anthocyanin accumulation in soybean hairy roots. This is a convenient, non-destructive, low cost, directly visual selection of transgenic hairy roots (Fan et al., 2020). Several efficient transformation protocols have been developed for studying functional genomics and root biology (Kereszt et al., 2007; Kuma et al., 2015; Chen et al., 2018a,c; Fan et al., 2020; Song et al., 2021).

#### Protoplast Transfection

The first genetic transformation of soybean protoplasts was achieved by electroporation by Lin et al. (1987). Dhir et al. (1992) was the first to report the transformation of immature cotyledon-derived protoplasts and regeneration of transgenic plants from calli derived from electroporation-transfected protoplasts. Protoplasts could be a good explant for transformation if an efficient regeneration system is established, especially since a large number of protoplasts can be transfected at a time and many forms of genetic materials such as DNA, RNA, and protein can be delivered. Unfortunately, protoplast transfection has not yet been conducted for soybean transgenic plant production. The main challenge is achieving protoplast regeneration, which has yet to be reported in soybean. Protoplast-based transfection has been mainly conducted to evaluate gene functions (Yi et al., 2010; Faria et al., 2011; Kidokoro et al., 2015; Xiong et al., 2019), screen promoters (Sultana et al., 2019), and validate vectors for GE (Sun et al., 2015; Demorest et al., 2016; Do et al., 2019; Patil et al., 2022). Recently, Wu and Hanzawa (2018) developed a method to isolate protoplasts from leaves of soybean seedlings and established a PEG-mediated transfection method that can achieve high transfection efficiency compared to other transient assays.

#### Agro-Infiltration

*Agrobacteria* can be infiltrated into the intercellular space of plant tissues to permit the delivery of genes from different organisms into plant genomes (Grimsley et al., 1986). Ever since this method was successfully established for soybean (King et al., 2015), it has been used for virus-induced gene silencing (VIGS) (Kim et al., 2016) and expression of hairpin RNA for RNAi against two-spot spider mites (Dubey et al., 2017).

#### *Agrobacterium tumefaciens*-Mediated Transformation

Except for stable transformation, *A. tumefaciens* is used to carry out transformation for soybean transient assay. Kun et al. (2017) established an *Agrobacterium*-mediated transient system using calli induced from hypocotyl explants. It has been successfully used in many specific assays including Western blot and Co-IP assay for protein analysis. The system is genotype-flexible and cost-saving. However, it takes a couple of months to complete the assay.

#### Electroporation

Electroporation is a technique that utilizes a high intensity electric pulse to create transient pores in the cell membrane, thereby facilitating the uptake of macromolecules such as DNA. Christou et al. (1988) conducted electroporation to deliver constructs into soybean calli and showed stable integration of genes but did not succeed in regenerating plants. Later, Chowrira et al. (1995) reported on electroporation of intact nodal meristems which avoided the soybean tissue culture process completely, but no transgenic plants have been recovered.

### Transformation Systems for Stable Transgenic Plants

**FIGURE 2:**
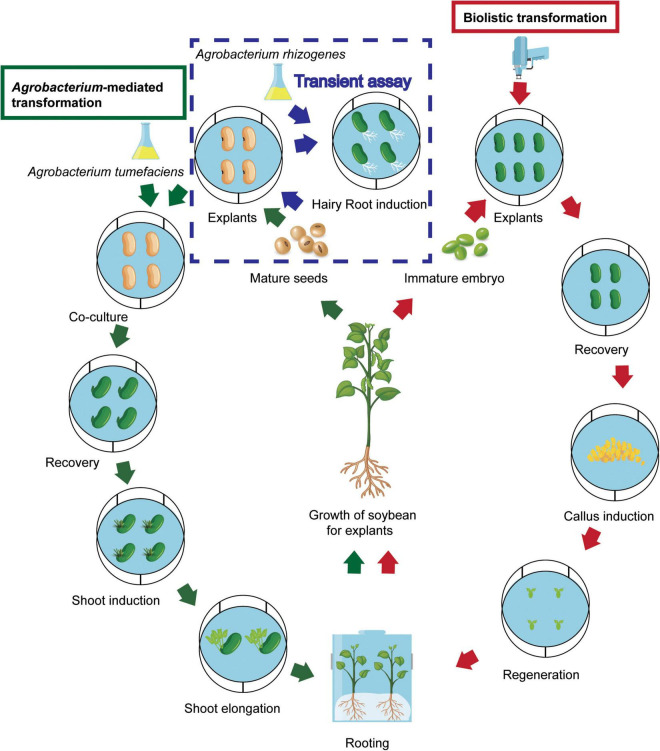
General procedure of *Agrobacterium*-mediated and biolistic soybean transformation. The route following the green arrows is the *Agrobacterium tumefaciens*-mediated transformation procedure. The route following the red arrows is the biolistic transformation procedure. The route following the blue arrows in the frame with broken blue line shows *Agrobacterium rhizogenes*-mediated transformation for transient assay.

*Agrobacterium*-mediated transformation and biolistic methods, and *in planta* transformation and protoplast transfection methods have been applied for generation of transgenic soybean plants. Among these methods, the *A. tumefaciens*-mediated and biolistic methods are the two major platforms for stable soybean transformation. The general transformation procedure of both methods is shown in Figure 2. The other methods mentioned above are used less because of relatively low efficiency and the specific technique and equipment required in these methods.

#### *A. tumefaciens*-Mediated Transformation

*A. tumefaciens*-mediated transformation of soybean was first initiated using cotyledonary nodes by Hinchee et al. (1988). Since the system was established based on regeneration of mature or immature seed explants, the simplicity and relatively high TF of the method have made it a favorite method for soybean. Relatively high efficient *Agrobacterium*-mediated transformation protocol has been gradually developed through improving factors such as using an appropriate *Agrobacterium* strain, a good explant, culture media with adequate antioxidant chemicals and combinations of appropriate plant growth regulators for a specific soybean genotype (reviewed in Yamada et al., 2012; Lee et al., 2013; Li et al., 2017; Table 1). Key elements of the progress are summarized in a later section. Main advantages of *Agrobacterium* transformation include relatively high ratio of single-copy gene insertion, relative simplicity of the transformation procedure, and low cost (Hwang et al., 2017). However, there is a limitation in delivery of genetic material. It delivers DNA plasmids but cannot deliver DNA fragments, RNAs, or proteins.

**TABLE 1:** Progress of soybean stable genetic transformation approaches for whole transgenic plants.

Method	Explant	Genotype	Selectable marker/agent	Physical treatment	Specific chemicals in medium	Agro-strain	Available TF (%)	References
*Agrobacterium*	Immature cotyledon	PI283332 and Peking	NptII/G418	Wounding	\	EHA101 and LBA4404	\	Parrott et al., 1989
		Jack	Hph/Hygromycin B	Wounding	AS	EHA105	0.03	Yan et al., 2000
		Jack, Williams, Ina, Macon, Dwight, and Rend	Hph/Hygromycin B	Wounding; orientation of explant (downward of the adaxial side)	AS	KYRT1	1.3 (1.1–1.7) (Jack)	Ko et al., 2003
	Mature cotyledonary node	Delmar, Maple Presto, and Peking	NptII/Kanamycin	\	\	A208	\	Hinchee et al., 1988
		28 genotypes	NptII/Kanamycin	Wounding and sonication	AS	KYRT1	1–2	Meurer et al., 1998
		A3237	Bar/Glufosinate	Wounding	AS, glutamine, and asparagine	EHA101 and EHA105	0.9	Zhang et al., 1999
		12 genotypes	NptII/Kanamycin	Wounding	\	A281, C58, ACH5, and EHA105	0.4 (one genotype)	Donaldson and Simmonds, 2000
		Bert	Bar/Glufosinate	Wounding	AS and L-cysteine	AGL1	2.1	Olhoft and Somers, 2001
		12 genotypes	Bar/Glufosinate, Hph/Hygromycin B, and NptII/Kanamycin	Wounding	AS, D-cysteine, and other thiol compounds	AGL1, LBA4404, GV3101, EHA105, and EHA101	\	Olhoft et al., 2001
		12 genotypes	Bar/Glufosinate	Wounding	AS, L-cysteine, DTT, asparagine, and glutamine	EHA101	2–6.3 (glufosinate) 0–2.9 (bialaphos)	Paz et al., 2004
		Williams82	Bar/Glufosinate	Wounding	AS and L-cysteine	EHA101	5.9	Zeng et al., 2004
		5 genotypes (Chinese soybean)	Hph/Hygromycin B	Wounding	AS, Silwet L-77, and L-cysteine, asparagine, and L-pyroglutamic acid	EHA105	3.8–11.7	Liu et al., 2008
		Kariyutaka	Bar/Glufosinate or Basta	Wounding (micro brush)	Silwet L-77	EHA105	4.4	Yamada et al., 2010
		PK416, JS90-41, Hara Soy, Co1, and Co2	Hph/Hygromycin B	Sonication and vacuum infiltration, wounding (hypodermic needle)	AS, DTT, L-cysteine, and sodium thiosulfate (STS)	LBA4404, EHA101, and EHA105	13.3–18.6	Arun et al., 2015
		JS-335	Bar/Glufosinate	Sonication and vacuum infiltration	AS, DTT, L-cysteine, and STS	EHA105	12.6 (10.5–16.2, J8335-bar);	Hada et al., 2018
		Jack and Zhonghuang 10	G2Epsps/Glyphosate	Sonication	Silwet L-77, AS, DTT, L-cysteine, and Na_2_S_2_O_3_	Ag10	2.9–5.7	Guo et al., 2015
		Jidou17	NptII/Kanamycin	Sonication	DDT, L-cysteine, sodium thiosulfate, and α-Aminooxyacetic acid	EHA105	\	Zhang et al., 2016
		7 genotypes	Bar/Glufosinate	Wounding	α-lipoic acid (α-LA), DTT, L-cysteine, AgNO_3_, glutamine, and asparagine	EHA101	14.7	Yang J. et al., 2016
	Half-seed	Bert	Hph/Hygromycin B	Wounding	AS, DTT, L-cysteine, and STS	LBA4404 and EHA105	16.4 (9.4–26.2 LBA4404); 14 (9.4–26.2 EHA105)	Olhoft et al., 2003
		Thorne, Williams, Williams79, and Williams82	Bar/Glufosinate	Wounding	AS, L-cysteine, and DTT	EHA101	3.8 (1.4–8.7)	Paz et al., 2006
		7 genotypes	Hph/Hygromycin B	Wounding (multi-needle)	AS, DTT, L-cysteine, and STS	LBA4404	\	Zhang and Xue, 2019
		5 US (Williams82) and 5 Chinese genotypes	Bar/Glufosinate	Wounding	AS, DTT, and L-cysteine	EHA105	0–6.71	Jia et al., 2015
		7 genotypes	Bar/Glufosinate	Wounding	L-cysteine and DTT	EHA105	0.5 (0–0.9)	Sato et al., 2007
		7 genotypes	Hph/Hygromycin B	Wounding (multi-needle)	AS, DTT, L-cysteine, and STS	LBA4404	\	Zhang and Xue, 2019
		5 US (Williams82) and 5 Chinese genotypes	Bar/Glufosinate	Wounding	AS, DTT, and L-cysteine	EHA105	0–6.71	Jia et al., 2015
		7 genotypes	Bar/Glufosinate	Wounding	L-cysteine and DTT	EHA105	0.5 (0–0.9)	Sato et al., 2007
		DS97–12	Hph/Hygromycin B	Sonication and vacuum infiltration	Polyamine (spermidine, spermine, and putrescine)	EHA105	29.3	Arun et al., 2016
		Williams82	Bar/Glufosinate	Wounding	AS, L-cysteine, and DDT	EHA101	1.0–3.5 (35s or NOS promoter)	Testroet et al., 2017
		8 genotypes	Bar/Glufosinate	Wounding	AS, DDT, STS, L-cysteine, AgNO_3_, L-asparagine, L-pyroglutamic acid, and L-ascorbic acid	EHA101	7.3–10.0	Li et al., 2017
		Jack, Williams82, Zigongdongdou, and Heihe27	Bar/Glufosinate	Wounding	DTT, AS, L-asparagine, and L-glutamine	EHA101	7.6 (2.6–11.1)	Chen et al., 2018b
		DS-9712	NptII/Kanamycin	Sonication and vacuum infiltration	AS and L-cysteine	EHA105	14.51	Hada et al., 2018
		PUSA 9712	Bar/Basta	\	SNP	EHA101	34.6	Karthik et al., 2020
		Maverick and 20 proprietary elite lines	Pat/Glufosinate	Wounding	L-asparagine and L-pyroglutamic acid	EHA101 and EHA105	18.7 (12.1–23.0)	Pareddy et al., 2020
	whole cotyledonary node	ZhongHuang13	NptII/Kanamycin	Wounding	L-cysteine	EHA105	23.1	Zhang et al., 2014
	Calluses induced from either cot-node	5 genotypes	Bar/Glufosinate	\	AS, DTT, L-cysteine, and STS	AGL1	1.3 (0.3–4.3)	Hong et al., 2007
	Hypocotyls	Heinong44	NptII/Kanamycin	\	AS, L-cysteine, DTT, AgNO_3_, and STS	EHA105	9.3	Wang and Xu, 2008
	Embryogenic cell suspension	Chapman	Hph/Hygromycin B	Sonication	AS	EHA105	\	Trick and Finer, 1998
	Embryogenic axes	P29T50, P33T50,93Y21, DM118, and 98C21	SpcN/Spectinomycin	Sonication	AS and DDT	*Ochrobactrum haywardense* H1	35	Cho et al., 2022
Biolistic method	Immature embryo axis	Williams82 and Mandarin Ottawa	NptII/Kanamycin	Electrical, arc-discharge gun/Gold particles		Plasmid DNA		Mccabe et al., 1988
		Williams82	NptII/Kanamycin	PDS 1000/Tungsten		Plasmid DNA		Sato et al., 1993
	Somatic embryogenic suspension	Fayette	Hph/Hygromycin B	DuPont Biolistics TM Particle Delivery System (Model BPG)/Tungsten particles		Plasmid DNA	0.4	Finer and Mcmullen, 1991
		Fayette	Npt II/G418	PDS 1000/Tungsten particles		Plasmid DNA	Four plants per bombarded flask	Sato et al., 1993
		Fayette	Hph/Hygromycin B	PDS 1000/Tungsten particles		Multiple plasmid DNA	\(co-transformation)	Hadi et al., 1996
		\	Hph/Hygromycin B	PDS 1000/Tungsten particles		Plasmid DNA	\(protocol)	Finer and Larkin, 2008
		\	Hph/Hygromycin B	PDS 1000/Tungsten particles		Plasmid DNA	\(protocol)	Finer, 2016
		93B86	Hph/Hygromycin B and Als/Chlorsulfuron	PDS 1000/Gold particles		Plasmid DNA and DNA fragment	\(targeted insertion)	Li et al., 2015
	Mature embryo axis	BR-16, Doko RC, BR-91, and Conquista	AHAS/Imazapyr	HPHMAS/Tungsten	\	Plasmid DNA	0.1–7.8	Aragão et al., 2000
		BR-16, BR-91, Celeste, Conquista, Doko RC, Nina, Indiana, and Itaipu	AHAS/Imazapyr	PDS1000/Tungsten		Plasmid DNA	≤0.2 (protocol)	Rech et al., 2008
		Conquista	AHAS/Imazapyr	HPHMAS/Tungsten	\	DNA fragments	0.8	Vianna et al., 2011
		INCASoy-36	Cp4epsps/Glyphosate	PDS 1000/Tungsten		Plasmid DNA	6	Soto et al., 2017
	Immature embryo	Maverick	Hph/Hygromycin B, DSM2/Glufosinate		Cold treatment and plasmolysis	Plasmid DNA	2–5.5 (hph) and 1–2.7 (DSM2)	Chennareddy et al., 2018

*(1) HPHMAS: The high-pressure helium-driven microparticle acceleration system. (2) \ means not available. (3) Protocol means the reference is a published protocol.*

#### Biolistic Transformation

Biolistic transformation, known as gene gun or particle bombardment, delivers small tungsten or gold particles coated with desired genes to target plant cells (Christou et al., 1988). Since an electrical-discharge gene gun was first used in soybean to regenerate a fertile transgenic plant (Mccabe et al., 1988), gene delivery to meristematic soybean cells by particle bombardment has been considered to be more genotype-flexible for transfer of foreign DNA into soybean (Homrich et al., 2012). Recently, embryogenic callus based biolistic method becomes more popular due to its relatively higher efficiency compared to other explants and its directly delivering way which meets the need for genome editing using RNA and RNPs editing reagents for recovery of DNA-free edited events. In comparison to the *A. tumefaciens*-mediated method, the biolistic method offers benefits with their capacity to transform organelles and deliver RNA, proteins, nanoparticles, dyes, and complexes to cells (Klein et al., 1987; Liang et al., 2017). The drawback is mainly high transgene copy and relatively high cost, and its application has been restricted in limited soybean genotypes because of unavailable meristematic explants. Compared to plasmid bombardment, utilization of specific constructs including linear minimal expression cassettes (MECs) in biolistic transformation enables the production of plants carrying much simpler patterns of transgene integration, which has been confirmed in plants such as wheat (Ismagul et al., 2018). The major progress in soybean biolistic transformation is presented in a later section and summarized in Table 1.

#### Other Stable Transformation Methods

##### *A. rhizogenes*-Mediated Transformation

Transgenic plants can also be produced by regeneration of hairy roots transformed with *A. rhizogenes.* Success of stable transformation has been reported in many plant species (Christey, 2001). In soybean, stable soybean transgenic plants were produced from hairy roots using primary-node explants infected by a disarmed *A. rhizogenes* strain SHA17 (Olhoft et al., 2007) and the several reports of targeted mutation events using genome editing also have been obtained from hairy roots through *A. rhizogenes*-mediated transformation (Curtin et al., 2011; Haun et al., 2014; Demorest et al., 2016). However, genotype inflexibility has been the main hurdle for using the method in soybean.

##### *In-Planta* Gene Transformation

This is an alternative method in which *Agrobacterium* is used to infect explants, but it does not involve *in vitro* culture and regeneration of plant cells or tissues (Kalbande and Patil, 2016), thereby reducing time and labor cost, and, most importantly, avoiding somaclonal variation occurrence during *in vitro* culture-mediated genetic transformation and regeneration. In soybean, an *Agrobacterium* suspension is directly injected into the ovary (Liu et al., 2009), axillary meristematic region of germinated seedling (Chee et al., 1989), or stigma in which exogenous DNA was introduced into cells *via* the “pollen-tube-pathway” (Hu and Wang, 1999). Transgenic events could be obtained from progeny seeds. Liu et al. (2009) reported the transfer of a minimal linear marker-free and vector-free smGFP cassette into soybean by pollen tube-mediated gene transfer. Mangena (2019) summarized the progress made in *in planta* transformation and formulated a simple protocol using *in planta Agrobacterium* injection of seedlings. Although this could be a tissue culture bypass method and attempts for new ways are made from time to time, its efficiency has been very low and it is often not repeatable. This method has not been widely used.

##### Transformation Using a New Bacterium

Recently a novel bacterium, *Ochrobactrum haywardense* H1 (Oh H1), was discovered and it is capable of efficient plant transformation (Cho et al., 2022). *Ochrobactrum* is able to host for *Agrobacterium*-derived *vir* and T-DNA and helps to deliver transgenes in soybean. Oh H1-8 generated high-quality transgenic events by single-copy, plasmid backbone-free insertion at frequencies higher than those of *Agrobacterium* strains. It achieved high transformation efficiency in several soybean genotypes, which can be up to 35%. The application of the new bacterium-mediated transformation in soybean needs to be evaluated further.

## Progress Made to Improve Soybean Transformation Over the Last Decade

Since 2010, increasing the transformation frequency (TF) has been the main focus for soybean transformation improvement. Several major factors affecting soybean TF based on *Agrobacterium*-mediated transformation have been identified, and progress has been made in establishing a high-throughput transformation system (Zhang et al., 2014; Arun et al., 2015, 2016; Yang X. F. et al., 2016; Li et al., 2017; Chen et al., 2018b; Karthik et al., 2020; Pareddy et al., 2020). Some confirmed positive elements in *Agrobacterium*-mediated transformation protocols have also been applied for enhancing soybean biolistic transformation (Table 1).

### *Agrobacterium*-Mediated Transformation

Soybean transgenic plant production still relies on *Agrobacterium*-mediated transformation (Figure 2 and Table 1). Recently, high TFs of over 10% have been obtained in more and more soybean genotypes using improved protocols (Zhang et al., 2014; Arun et al., 2015, 2016; Yang X. F. et al., 2016; Li et al., 2017; Chen et al., 2018b; Karthik et al., 2020; Pareddy et al., 2020). The enhancement of TF is based on changes in several factors, including explant, selectable marker, and culture medium composition such as antioxidants, of these protocols (Table 1).

#### Adjustment of Infection Method and Improving Regeneration

Reducing the explant tissue browning and necrosis caused by *Agrobacterium* enhances construct delivery and regeneration of transformed cells. Changing the ways for preparation of *Agrobacterium* infection solutions and co-cultivation media, and modifying infection methods can achieve this goal and eventually increase transformation efficiency. Addition of antioxidants such as dithiothreitol (DTT) in infection solutions and extending co-cultivation time to 5 days achieved an infection efficiency of more than 96% and, hence, increased TF (Li et al., 2017). Infection solutions prepared with a two-round overnight culture of *Agrobacterium* using AB minimal media in second round culture significantly increased transformation frequency in comparison with the culture using normal YEP medium (Pareddy et al., 2020). It was also found to be beneficial to *A. tumefaciens* infection when the co-cultivation temperature for soybean transformation was set to 23°C under dim light (Yang X. F. et al., 2016). The same group also demonstrated to alleviate explant necrosis and significantly improve the transformation efficiency when antioxidants alone such as α-lipoic acid (α-LA, 0.12 mM) and silver nitrate (AgNO_3_, 20 μM), or combinations of antioxidants such as L-cysteine (1 mM) + DTT (3.3 mM) + AgNO_3_ (20 μM), and L-cysteine (1 mM) + DTT (3.3 mM), were added in the solid co-cultivation medium. For improving regeneration, it was found that adding 6-benzylaminopurine (BAP) in a germinating medium could significantly increase regeneration efficiency, which led to enhancement of TF; the optimal BAP concentration for shoot formation was 0.5 mg/L (Zhang et al., 2014). More examples are presented in Table 1.

#### Genotype Effect and Explant Choice

In the tissue culture-based transformation process, the composition of culture media and susceptibility of selected explants to *Agrobacterium* influence soybean transgenic frequency. A highly efficient *in vitro* culturing system and regeneration of cells susceptible to *Agrobacterium* are prerequisites for a reliable transformation protocol. Until now, the TF for most tested genotypes of soybean has remained quite low at a level mostly below 5% when conducting *Agrobacterium*-mediated transformation [summarized in Yamada et al. (2012); Jia et al. (2015), and Li et al. (2017); Table 1]. Since 2000, many research groups have used model soybean varieties such as Jack, Bert, and Williams serials and other specific genotypes because of their amenability to transformation (Olhoft and Somers, 2001; Olhoft et al., 2001, 2003; Paz et al., 2004, 2006; Zeng et al., 2004; Luth et al., 2015). Recently, soybean transformations with high TFs have been reported using specific genotypes. For example, it was claimed 23.1% with Zhonghuang13 (Zhang et al., 2014) and an average of 14% TF for a local Indian genotype, DS-9712 (Hada et al., 2018). Improvement based on *Agrobacterium*-mediated soybean transformation has been made to expand target genotypes from conventional model varieties to many elite varieties (Ko et al., 2003; Yi and Yu, 2006; Sato et al., 2007; Song et al., 2013; Arun et al., 2015; Pareddy et al., 2020). For example, over 5% TF for more than 10 varieties was achieved with a robust protocol (Pareddy et al., 2020).

**FIGURE 3:**
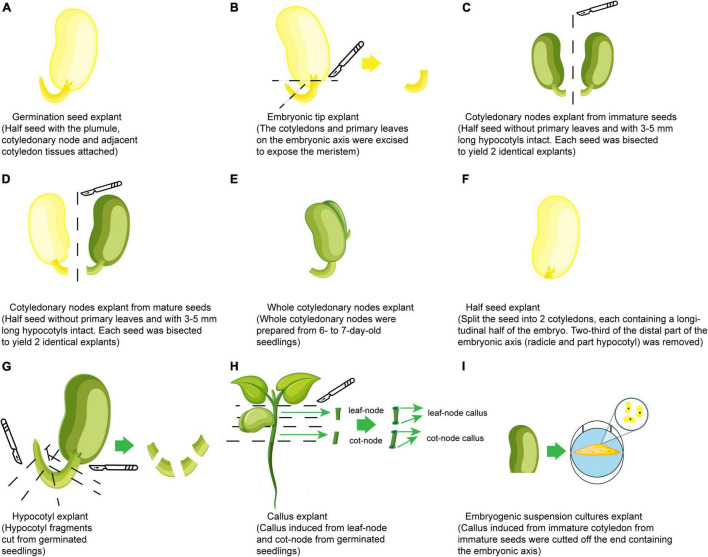
Types of explants used for soybean transformation. **(A)** Germination seed. **(B)** Embryonic shoot tips. **(C)** Cotyledonary nodes from immature seeds. **(D)** Cotyledonary nodes from mature seeds (yellow cotyledon means use cotyledon directly from mature seed; the green one means use mature cotyledon after germination under light). **(E)** Whole cotyledonary node. **(F)** Half-seed. **(G)** Hypocotyl. **(H)** Callus induced from geminated seedlings. **(I)** Embryogenic suspension cultures.

Since Hinchee et al. (1988) obtained transgenic events, the cotyledonary node of mature seeds has been the most favorite explant used for *Agrobacterium*-mediated soybean transformation using many other explants such as embryonic tips and calli (Figure 3). Cotyledonary node regions have axillary meristems at the junction between cotyledon and hypocotyl, which can proliferate and regenerate by the formation of multiple adventitious shoots on a culture medium containing cytokinin. Successful transformation has been achieved using similar organogenesis from various explants, which include germination seeds (Chee et al., 1989), embryonic shoot tips (Martinell et al., 2002; Liu et al., 2004), cotyledonary nodes from immature seeds (Parrott et al., 1989; Yan et al., 2000; Ko et al., 2003), cotyledonary nodes from mature seeds (Meurer et al., 1998; Zhang et al., 1999; Donaldson and Simmonds, 2000; Olhoft and Somers, 2001; Olhoft et al., 2001; Paz et al., 2004; Zeng et al., 2004; Liu et al., 2008), half-seeds (Paz et al., 2006; Pareddy et al., 2020), whole cotyledonary nodes (Zhang et al., 2014) and hypocotyls (Dan and Reichert, 1998; Liu et al., 2004; Wang and Xu, 2008), and other explants with different regeneration procedures such as calli induced from geminated seedlings (Hong et al., 2007) and embryogenic suspension cultures (Trick and Finer, 1998). However, successful and repeatable production of transgenic soybean *via Agrobacterium*-mediated transformation has mainly been based on protocols with explants containing cotyledonary nodes from young seedlings and imbibed mature seeds (Zhang et al., 1999; Olhoft et al., 2003; Paz et al., 2006). Recently, half-seeds have gradually become the trend for explants since (Paz et al., 2006) their first use, because half-seed explants possess advantages to have more nutrition supply for shoot regeneration compared to cotyledonary nodes and to be prepared within a short time (less than 1 day) due to using imbibed seeds, which reduces the period of total regeneration and labor cost. Based on descriptions of explants in several reports (Paz et al., 2006; Pareddy et al., 2020), half-seed, whole cotyledon, and split seed explants can now be put under the same category of half-seed explants. Obtaining TFs of over 10% for soybean with half-seed explants have been demonstrated in many reports (Zhang et al., 2014; Arun et al., 2016; Li et al., 2017; Chen et al., 2018b; Hada et al., 2018) (Table 1). The highest TF of 34.6% has been obtained using these explants together with nitric oxide treatment in a co-cultivation medium in the protocol made by Karthik et al. (2020). Some specific explant treatments such as sonication in combination with vacuum infiltration, sonication in combination with surfactant, or just sonication (Mariashibu et al., 2013; Arun et al., 2015; Guo et al., 2015; Zhang et al., 2016; Hada et al., 2018), and pre-wounding with a multi-needle consisting of 30 thin fibers (Xue et al., 2006) or a micro-brush (Yamada et al., 2010) were also used before *Agrobacterium* infection to increase infection rate and TFs, because these treatments facilitate the penetration of *Agrobacterium* into plant tissues and increase the contact between plant cells and the bacterium, and stimulate the infection ability of the bacterium, which leads to T-DNA transfer into plant cells.

#### Addition of Antioxidants in Medium

Antioxidants, in general, are known to reduce pathogen-induced programed cell death (Mittler et al., 1999). These include inhibitors of polyphenol oxidases (PPOs) and peroxidases (PODs) through the action of their thiol group, such as compounds L-cysteine, DTT, and sodium thiosulfate. They are commonly used to reduce enzymatic browning in food processing caused by deposition of tannins (Nicolas et al., 1994; Ghidelli et al., 2014). Polyvinylpyrrolidone (PVP), DTT, L-cysteine, glutathione, α-LA, L-ascorbic acid, and citric acid have been confirmed to decrease tissue necrosis of explants used for *Agrobacterium*-mediated transformation (Barampuram and Zhang, 2011). Either one or more than 2 of the chemicals have been used in soybean transformation (Olhoft and Somers, 2001; Olhoft et al., 2003; Paz et al., 2004; Yi and Yu, 2006; Liu et al., 2008). L-cysteine and DTT have been frequently used in soybean transformation since its first use by Olhoft et al. (2001). Reports clearly showed that there was less browning on the cut and damaged surfaces of the hypocotyl, cotyledon node region, and on the cotyledon of explants, which increased the TF of stable transformations (Olhoft et al., 2001, 2003; Paz et al., 2004; Li et al., 2017). A high average TF of 12.7% resulted from the combination of L-cysteine and DTT, which was significantly greater than that of either L-cysteine or DTT alone (Olhoft et al., 2003). The positive effect has been continuously confirmed in recent reports (Table 1). Another type of antioxidants is a group of sulfur-containing compounds involved in several multienzyme complexes such as α-LA. These include pyruvate dehydrogenase, α-ketoglutarte dehydrogenase, branched-chain ketoacid dehydrogenase, and glycine decarboxylase (Dan et al., 2009). Adding the antioxidant α-LA in a co-cultivation medium could increase transient GUS expression and increased the percentage of shoot induction (Yang X. F. et al., 2016). In this report, 0.12 mM α-LA was found to be the most useful for alleviating browning and necrosis. Other antioxidants conventionally used in plant tissue culture, such as ascorbic acid, PVP, and citric acid, may promote soybean transformation efficiency, but their roles have not yet been made clear (Li et al., 2017). Plant hormone-like antioxidants such as sodium nitroprusside (SNP), a nitric oxide (NO) donor, play varied roles in growth and development of plants. Nitric oxide is involved in cell metabolism and morphogenesis and acts as a signaling molecule in response to various biotic and abiotic stresses (Verma et al., 2020), and can alleviate abiotic stress threat in plants reacting quickly with ROS. SNP significantly enhanced regeneration and development rate of soybean plants (Karthik et al., 2019); addition of SNP also significantly increased soybean TF by up to 34.6% with the half-seed method (Karthik et al., 2020).

#### Addition of Other Chemicals in Culture Medium

Except for antibiotics, chemicals related to host defense response, ethylene inhibitors, surfactants, demethylating reagents, polyamines, and antagonist α-aminooxyacetic acid (AOA) are proved to have a positive effect on improving TF. L-glutamine and L-asparagine are types of chemicals that weaken host defense responses. It has been reported that the addition of L-glutamine into a culture medium alone or in combination with a cold shock pretreatment could enhance *Agrobacterium* transformation efficiency (Zhang et al., 2013). Although the mechanism is still not clear, L-glutamine could play a role in lessening host defense responses by attenuating the expression of certain pathogenesis-related genes (PRs), and potentially improve the efficiency of *Agrobacterium*-mediated plant transformation (Zhang et al., 2013, 2014). It was demonstrated that TF was significantly increased in soybean when additional L-glutamine or L-asparagine alone, or both of them were added in all culture media (Chen et al., 2018b). The TF was 8.8 ± 1.5 (L-glutamine), 5.9 ± 2.1% (L-asparagine), 11 ± 0 (both), and 3.5 ± 2.4% (without any one of them). Ethylene inhibitors such as AgNO_3_ have a positive effect on transformation. It has been reported that Ag^+^ interferes with the binding of ethylene receptor sites and helps reduce ethylene production by promotion of polyamine biosynthesis (Roustan et al., 1990). The main function of AgNO_3_ is to eliminate the potential danger to plant cells and tissues in liverwort caused by ethylene (Beyer, 1979). It has already been confirmed to promote somatic embryo production and shoot regeneration in wheat and maize (Carvalho et al., 1997; Fernandez et al., 1999). This effect has been proved to improve soybean TF (Olhoft et al., 2004; Li et al., 2017). A nearly 10% TF with genotype Heilong44 was reported when BAP and AgNO_3_ were added into a culture medium (Wang and Xu, 2008). Surfactants such as SilwetL-77 and pluronic acid F68 also increase TF, which initially showed to enhance T-DNA delivery in wheat *Agrobacterium*-mediated transformation when added into an inoculation medium (Cheng et al., 1997). This was also confirmed in soybean transformation. It was reported that adding SilwetL-77 to an infection medium coupled with hygromycin-based selection strategies led to transformation efficiencies ranging from 3.8 to 11.7% in Chinese soybean varieties (Liu et al., 2008). SilwetL-77 has been frequently used to increase soybean TF (Yamada et al., 2010; Guo et al., 2015). Surfactants may enhance T-DNA delivery by aiding *A. tumefaciens* attachment and/or by elimination of certain substances that inhibit *A. tumefaciens* attachment (Opabode, 2006). Polyamines enhance plant cell differentiation, induce totipotency, and increase cell division (Rakesh et al., 2021). Addition of polyamines in the plant transformation process leads to *vir* gene induction and T-DNA transfer, and increases transformation efficiency (Kumar and Rajam, 2005). As high as 29.3% TF in soybean has been achieved by addition of spermidine, spermine, and putrescine in a culture medium compared with its counterparts (14.6%) and with respective plant growth regulator (PGR) alone (Arun et al., 2016). Demethylating reagents commonly applied in epigenetic research such as 5-azacytidine (5-Azac), significantly improve the transient transfection efficiency and transgene expression level in low-efficiency genotypes. Treatment with 5-Azac improved the shoot regeneration efficiency in low-efficiency genotypes during the process of *Agrobacterium*-mediated soybean transformation. This indicates that lower methylation level in transgenes contributed to enhance shoot regeneration in *Agrobacterium*-mediated soybean transformation (Zhao et al., 2019b). Antagonist AOA relieves the structural membrane barriers of *Agrobacterium* entering cells, hinders the perception of intercellular signal transmission, and thus effectively alleviates defense responses and increases the susceptibility of cells to *Agrobacterium* infection. Combined use of AOA and sonication treatments (novel method) greatly improved T-DNA delivery efficiency in soybean (Zhang et al., 2015, 2016).

#### Refining Selection Agents

The most frequently used selectable markers in both the somatic embryogenesis- and organogenesis-based soybean transformation methods are genes conferring resistance to herbicides or antibiotics so as to reduce escape rate significantly. The selectable markers include *bar* and *pat* genes conferring resistance to phosphinothricin, the active ingredient in BASTA and bialaphos herbicides (Zhang et al., 1999; Olhoft and Somers, 2001; Olhoft et al., 2001; Paz et al., 2004; Testroet et al., 2017; Pareddy et al., 2020), EPSPS (5-enolpyruvylshikimate-3-phosphate synthase) genes conferring resistance to the herbicide glyphosate (Martinell et al., 1999; Clemente et al., 2000; Yao, 2001; Guo et al., 2015; Xiao et al., 2019), and the *nptII* gene conferring resistance to the antibiotics kanamycin (Homrich et al., 2012) and *hph or hpt II* (hygromycin phosphotransferase) genes conferring resistance to hygromycin B (Yan et al., 2000; Ko et al., 2003; Olhoft et al., 2003; Liu et al., 2008). Recently, *Hph* and *Bar* or *Pat* have been proven to be the most favorite selectable markers (Table 1). An average transformation frequency as high as 29.3% was achieved with the half-seed (Arun et al., 2016) and 13.3–18.6% with cotyledonary node explant (Arun et al., 2015) employing hygromycin B selection, which is better than or comparable with that by Olhoft et al. (2003). Glufosinate has also been used as a selection agent based on the *bar* or the *pat* gene and initially had less than 10% TF in soybean transformations involving the half-seed (Paz et al., 2006) and embryo tip (Dang and Wei, 2007) explants. Recently TFs of over 10% have been obtained with cotyledonary nodes (Hada et al., 2014; Yang X. F. et al., 2016) and half-seeds (Li et al., 2017; Chen et al., 2018b; Pareddy et al., 2020). A 34.6% TF was reported using a protocol with addition of sodium nitroprusside (SNP) when Basta was sprayed for selection (Karthik et al., 2020). Since an *epsps*/glyphosate selection based protocol is established (Martinell et al., 1999), glyphosate has gradually been incorporated into transformation as a selectable agent and has shown its beneficial side for high stringency. In order to quickly and efficiently screen glyphosate-tolerant events, a rapid and convenient spotting method was established for screening regenerated glyphosate-tolerant T_0_ plantlets (Guo et al., 2020a). In this report, an optimized *Agrobacterium*-mediated soybean transformation system with rapid and effective selection of transformed cells was developed, with TFs ranging from 2.9 to 5.6%. Especially, 96% regenerated T_0_ plantlets showed clear tolerance to glyphosate and their transgenic nature were confirmed by molecular analysis. Spectinomycin was also used as a selective agent to obtain transgenic soybean when the aminoglycoside-3′′-adenylyltransferase gene (aadA) was used as a selectable marker (Martinell et al., 2002). The spectinomycin selection protocol demonstrated higher frequency of transformation, a shorter period of time needed to complete each protocol, and lower cost compared with the glyphosate selective protocol. Soybean transformation using a GFP as a detection marker was also reported, and transgenic plants could be identified at an early stage, although the frequency was not high (2.5%) (Yang S. et al., 2019). Combined with a normal selectable marker and a selection agent, GmFAST (fluorescence-accumulating seed technology) has recently been developed to identify homologous transgenic seeds. It is a marker composed of a soybean seed-specific promoter coupled to the OLE1-GFP gene, which encodes a GFP fusion of the oil-body membrane protein OLEOSIN1 of *Arabidopsis thaliana* and is a time-saving and efficient method to produce homologous transgenic events (Iwabuchi et al., 2020).

Generally, the efficiency of *Agrobacterium*-mediated transformation in soybean has been enhanced by improving both *Agrobacterium* infection and explant regeneration. Addition of antioxidants such as DDT, L-cysteine, and NO in a co-cultivation medium and some infection-assisting specific chemicals including surfactants and AgNO_3_, and some regeneration-promoting elements such as polyamines (Table 1), plays an important role in the improvement. All of the measures have facilitated *Agrobacterium* to transform the meristematic region of soybean explants. Recently, a specific *Agrobacterium*-mediated protocol was reported, which conducted bombardment to make wounding and reduce the lab work time to only 2 days in the transformation process and kept the rest time to grow T_0_ plants in a glasshouse (Paes de Melo et al., 2020). Transgenic events were screened using a swab to spread glufosinate solution on leaves of putative events and the TF reached nearly 10%. This method avoided many tissue culture steps and may be a cost-saving protocol.

### Biolistic Transformation

Since Mccabe et al. (1988) reported the first transgenic soybean plant using the biolistic method, many reports of soybean biolistic transformation have been published and the development of this method in the first 25 years has been reviewed by Homrich et al. (2012); Lee et al. (2013), Mariashibu et al. (2013), and Mangena et al. (2017). Initially, meristems of soybean tissues as the target tissue were used for bombardment such as embryonic axes of immature and mature seeds (Sato et al., 1993; Aragão et al., 2000; Rech et al., 2008; Soto et al., 2017). In later studies, somatic embryos (Finer and Mcmullen, 1991; Finer and Larkin, 2008; Finer, 2016) were the most frequently used explants for biolistic transformation. However, chimeric transgenic plants were produced because of multiple cell layers (L1, L2, and L3) in the original apical meristem of soybean (Christou, 1990; Christou and Mccabe, 1992). Fortunately, using secondary somatic embryos and new selective markers such as EPSPS has eliminated transgenic chimeras (Sato et al., 1993; Martinell et al., 1999). Somatic embryo regeneration and proliferation were initiated either on semi-solid media (Parrott et al., 1989) or liquid suspension culture media (Finer and Nagasawa, 1988). Co-transformation of multiple plasmids or multiple gene inserts in same constructs with selectable markers has been achieved (Hadi et al., 1996; Li et al., 2015). Since 2010, factors such as explant type, abiotic stress treatment, selectable marker, and tissue culture method have been the main focus to improve biolistic transformation TF, and reliable protocols for the biolistic method with embryogenesis-based explant have been developed to produce a reasonable number of transgenic plants (Table 1). For example, a TF of up to 6% was achieved with *cp4epsps* as selectable marker when embryonic axes of mature seeds of the INCASoy-36 Cuban cultivar were bombarded (Soto et al., 2017). Chennareddy et al. (2018) combined an immature half-seed explant with an intact embryonic axis, cold and plasmolysis pre-treatment, and a specific somatic embryogenic callus regeneration medium in their protocol. They achieved 5% TF with HPH/hygromycin selection and 2.7% with DSM2/glufosinate selection. A selection system using NPTII/G418 was developed for a biolistic-transformed embryogenic callus rather than the most used HPH/hygromycin system and similar TF in comparison with the HPH system was obtained (Itaya et al., 2018). The current status is that soybean biolistic transformation still relies on an embryogenic callus, since it is the prerequisite for establishing a robust transformation system for a specific genotype. Selection for the amenability of an embryogenic callus induced from local elite varieties (genotypes) is the main focus (Joyner et al., 2010; Abbasi et al., 2016; Islam et al., 2017; Raza et al., 2020). An improved biolistic soybean transformation protocol was published using an embryogenic callus induced from an immature cotyledon explant (Finer, 2016), which is a robust one and can produce quite a lot of transgenic plants within 6–9 months.

## Recent Applications of Soybean Transformation for Trait Improvement

Transgenic technology has been used to improve soybean agronomic traits, which include yield component, grain quality, and biotic and abiotic stress tolerance, and economic traits such as oil and biofuel quality, and specific chemical content in seed for human health, and other traits. Trait improvements through forward and reverse genetic approaches in the last 5 years are summarized in Table 2; i.e., downregulation of the pyruvate dehydrogenase kinase gene *GmPDHK* through RNAi made an average of 42.2% protein content in seeds of transgenic plants, which is significantly increased compared with the non-transgenic control (Jones et al., 2020). Soybean seeds with linolenic acid content in excess of 50% of the total oil have been generated by increasing the expression of the *FAD3* gene, which encodes the enzyme that converts linoleic acid to linolenic acid (Yeom et al., 2020). Overexpressing the *GmmiR156b* (Squamosa promoter-binding protein-like, SPL) gene in soybean and transgenic plants produced significantly increased numbers of long branches, nodes, and pods that exhibited increased 100-seed weight, resulting in a 46–63% increase in yield per plant and no significant impact on plant height in a growth room or under field conditions (Sun et al., 2019). Stable *GmMYB14*-overexpressing (*GmMYB14*-OE) transgenic soybean plants demonstrate semi-dwarfism and a compact plant architecture associated with decreased cell size, causing decreased plant height, internode length, leaf area, leaf petiole length, and leaf petiole angle, and improved yield in high density and drought tolerance under field conditions (Chen et al., 2021b). Salt-tolerant transgenic soybean and its applications in field are summarized in a review (Cao et al., 2018). Resistance to soybean cyst nematode (SCN; *Heterodera glycines*) in stably transformed soybean plants is enhanced by downregulation of the *HgY25* and *HgPrp17* genes, which are related to reproduction and fitness (Tian et al., 2019). Overexpression of *PAC1* and *GmKR3*, a TIR–NBS–LRR-type R gene, can increase multiple virus resistance in transgenic soybean and, thus, provide an efficient control strategy against RNA viruses such as SMV, BCMV, WMV, and BPMV (Xun et al., 2019). Overexpression of *GmDR1* [*Glycine max* disease resistance 1 (*Glyma.10G094800*)] led to enhanced resistance not only against *F. virguliforme* but also against spider mites (*Tetranychus urticae*, Koch), soybean aphids (*Aphis glycines*, Matsumura), and SCN (Ngaki et al., 2021). Many types of herbicide-resistant transgenic soybean, such as glyphosate-resistant, dicamba-, and 2,4-D-resistant, are grown widely in the United States (Nandula, 2019). Transgenic soybean plays an important role in soybean production worldwide now, and transgenic soybean covers 50% of the global transgenic crop area, occupying 94.1 million ha (Nandula, 2019). Therefore, a better soybean transformation system is the base for soybean improvement through transgenic technology.

**TABLE 2:** Summary of transgenic approaches for soybean trait improvement and functional genomics in the last 5 years.

Target traits	Transgene	Source of gene	Delivery method	Effect on trait or function	Genotype	References
**Seed components and quality**						
Seed protein and amino acid	*Zm*δ*-zeins* and *Zm*γ*-zein*	*Z. mays*	*A. tumefaciens*	Increase 27% the methionine content	Williams82	Kim and Krishnan, 2019
	*Zm*β*-zein*	*Z. mays*	*A. tumefaciens*	Increase 15% the methionine content	Jack	Guo et al., 2020b
	*GmPDHK*	*G. max*	Biolistic method	Increase average 42.2% protein content	Jack	Jones et al., 2020
	*Glyma.10G38760a*	*G. max*	*A. tumefaciens*	Increase sulfur amino acid content	Maverick	Kim et al., 2020
Oil	*GmFAD2-1B*	*G. max*	*A. tumefaciens*	Increase oleic acid content	Williams82	Yang J. et al., 2018
	*GmSDP1-1*	*G. max*	*A. tumefaciens*	Increase oil content	Kariyutak	Kanai et al., 2019
	*PfFAD3-1*	*P. fendleri*	*A. tumefaciens*	Increase α-linolenic acid production	Kwangankong	Yeom et al., 2020
	*GmOLEO1*	*G. max*	*A. tumefaciens*	Increase 10.6% seed oil content and enriched smaller OBs	Williams82	Zhang et al., 2019a
	*GmWRI1b*	*G. max*	*A. tumefaciens*	Increases total seed oil production	**\**	Guo et al., 2020c
	*Glyma.13G30950*	*G. max*	*A. tumefaciens*	Increase seed pods and oil production	Kariyutaka	Iwabuchi et al., 2020
	*GmDGAT2A*	*G. max*	*A. tumefaciens*	Increase oil production and α-linoleic acid content	P03	Jing et al., 2021
	*GmZF392*	*G. max*	*A. tumefaciens*	Increase seed oil accumulation	Jack	Lu et al., 2021
	*GmWRI1a*	*G. max*	*A. tumefaciens*	Increase seed oil content	Dongnong50	Wang Z. et al., 2022
	*AhDGAT3*	*A. hypogaea*	*A. tumefaciens*	Increase oleic acid and total fatty acid	Jack	Xu et al., 2022
Bioreactor	*rhBMP2*	*H. sapiens*	Biolistic method	Result in production of bone morphogenetic protein BMP2	BRS16	Queiroz et al., 2019
	The lunasin gene	*G. max*	*A. tumefaciens*	Result in production of bioactive lunasin peptide	**\**	Hao et al., 2020
	The *hIFN*-γ gene	*H. sapiens*	*A. tumefaciens*	Result in production of human IFN-γ protein	Williams	Mehrizadeh et al., 2021
Phytate content	*GmIPK2*	*G. max*	*A. tumefaciens*	Result in production of low phytate	Pusa-16	Punjabi et al., 2018
	*GmMIPS1*	*G. max*	*A. tumefaciens*	Regulate phytate biosynthesis	DS-9712	Kumar et al., 2019
	*EcMappA*	*E. coli*	*A. tumefaciens*	Result in production of a thermostable phytase	Wandou-28	Zhao et al., 2019c
Specific chemical compounds	*ZmGB1*	*Z. mays*	*A. tumefaciens*	Increase glycinebetaine content	A5403, A4922, A3469, and A3244	Castiglioni et al., 2018
	*GmCHI1A*	*G. max*	*A. tumefaciens*	Increase seed isoflavones	DT2008	Nguyen et al., 2020
	*GmMATE1*	*G. max*	*A. tumefaciens*	Increase seed isoflavones	C08 and W05	Ng et al., 2021
	*GmMYB176* and *GmbZIP5*	*G. max*	*A. rhizogenes*	Increase seed isoflavones	Harosoy63	Anguraj Vadivel et al., 2021
**Agronomic traits**						
Seed yield and plant biomass	*GmPT7*	*G. max*	*A. tumefaciens*	Increase symbiotic N_2_ fixation and yield	HN66	Chen et al., 2019b
	*GmmiR156b*	*G. max*	*A. tumefaciens*	Improve the shoot architecture and yield	Williams82	Sun et al., 2019
	*psNTP9*	*P. sativum*	*A. tumefaciens*	Increase soybean yield	Williams82	Veerappa et al., 2019
	*GmWRI1b*	*G. max*	*A. tumefaciens*	Improve plant architecture and associated yield parameters, and increases total seed oil production	**\**	Guo et al., 2020c
	*HaHB4*	*H. annuus*	*A. tumefaciens*	Enhance drought tolerance with yield reduced	Williams82	Ribichich et al., 2020
	*GmMYB14*	*G. max*	*A. tumefaciens*	Enhance high-density yield and drought tolerance	Tianlong1	Chen et al., 2021b
	*ZmSOC1*	*Z. mays*	*A. tumefaciens*	Increase soybean yield	Jack	Han et al., 2021
	*GmFULa*	*G. max*	*A. tumefaciens*	Increase soybean yield	Zigongdongdou	Yue et al., 2021
	*GmHSP17.9*	*G. max*	*A. rhizogenes*	Increase nodule number, nodule fresh weight, and seed yield	Williams82	Yang et al., 2022
Plant architecture	*GmIDL2a and GmIDL4a*	*G. max*	*A. rhizogenes*	Increase the lateral roots densities of the primary roots	XIAOLIDOU	Liu C. et al., 2018
	*GmmiR156b*	*G. max*	*A. tumefaciens*	Improve the shoot architecture and yield	Williams82	Sun et al., 2019
	*GmYUC2a*	*G. max*	*A. rhizogenes*	Delay nodule development and a reduced number of nodules	Williams82	Wang et al., 2019c
	*GmGASA32*	*G. max*	*A. rhizogenes*	Increase plant height	Williams82	Chen et al., 2020b
	*GmWRI1b*	*G. max*	*A. tumefaciens*	Improve plant architecture and associated yield parameters, and increases total seed oil production	**\**	Guo et al., 2020c
	*Glyma.13G30950*	*G. max*	*A. tumefaciens*	Increase seed pods and oil production	Kariyutaka	Iwabuchi et al., 2020
	*GmPIF4b*	*G. max var.* Bragg	*A. tumefaciens*	Affect plant morphology and accelerating reproductive phase transitions	Bragg	Arya et al., 2021
	*AtBIC1*	*A. thaliana*	*A. tumefaciens*	Increase plant height	Kwangankong	Cho et al., 2021
	*GmDIR27*	*G. max*	*A. tumefaciens*	Increase pod dehiscence	Williams82	Ma X. et al., 2021
	*GA2ox8A* and *GA2ox8B*	*G. max*	*A. tumefaciens*	Decrease trailing growth and shoot length	W05	Wang et al., 2021d
	*GmGAMYB*	*G. max*	*A. tumefaciens*	Promote flowering and increase plant height	DongNong50	Yang et al., 2021
	*GmBICs*	*G. max*	*A. tumefaciens*	Increase stem elongation	TianLong1	Mu et al., 2022
Iron, nitrogen, and phosphorus use efficiency	*GmbHLH57* and *GmbHLH300*	*G. max*	*A. rhizogenes*	Enhance Fe uptake and increase the Fe content in plants	Williams82	Li et al., 2018
	*GmPT7*	*G. max*	*A. tumefaciens*	Enhance symbiotic N_2_ fixation and yield	HN66	Chen et al., 2019b
	*GmWRI1s*	*G. max*	*A. rhizogenes*	Increase nodule numbers	Tianlong1	Chen et al., 2020a
	*GmPAP12*	*G. max*	*A. rhizogenes*	Increase nodule numbers	Williams82	Wang et al., 2020d
	*GmAAP6a*	*G. max*	*A. tumefaciens*	Enhance tolerance to low nitrogen and improve seed nitrogen status	Tianlong1	Liu et al., 2020
	*GmMDH12*	*G. max*	*A. tumefaciens*	Decrease nodule size and mediates malate synthesis	YC03-3	Zhu et al., 2021
	*GmNMHC5*	*G. max*	*A. tumefaciens*	Increase nodulation	Jack	Wang W. et al., 2022
	*GmNINs*	*G. max*	*A. rhizogenes*	Decrease nodule numbers	Williams82 and Huachun6	Fu et al., 2022
	*GmD27c*	*G. max*	*A. rhizogenes*	Increase nodule numbers	Tianlong1	Rehman et al., 2022
	*GmSPX8*	*G. max*	*A. rhizogenes*	Increase nodule number, nodule fresh weight, and nitrogenase activity	Zhonghuang15	Xing et al., 2022
	*GmHSP17.9*	*G. max*	*A. rhizogenes*	Increase nodule number, nodule fresh weight, and seed yield	Williams82	Yang et al., 2022
	*EsPHT1;4*	*E. salsugineum*	*A. tumefaciens*	Increase tolerance to low phosphorus stress	YD22	Yang et al., 2020b
	*GmETO1*	*G. max*	*A. tumefaciens*	Enhance Pi deficiency tolerance	NN94156 and Bogao	Zhang H. et al., 2020
Flowering time	*GmFT1a* and *GmFT2a/5a*	*G. max*	*A. tumefaciens*	*GmFT1a* and *GmFT2a/5a* have opposite roles in controlling flowering	Zigongdongdou and Heihe27	Liu W. et al., 2018
	*GmFT2b*	*G. max*	*A. tumefaciens*	Promote flowering	Jack	Chen et al., 2020c
	*GmGAMYB*	*G. max*	*A. tumefaciens*	Promote flowering and increase of plant height	DongNong50	Yang et al., 2021
	*E1* (*Glyma06g23026*)	*G. max*	*A. tumefaciens*	Promote flowering	Zigongdongdou	Liu et al., 2022a
**Abiotic and biotic traits**						
Nematode resistance	*HgY25*	*H. glycines*	Biolistic method	Enhance resistance to soybean cyst nematodes	Jack	Tian et al., 2019
	*BtCry14Ab*	*B. thuringiensis*	Biolistic method	Enhance resistance to soybean cyst nematodes	Jack	Kahn et al., 2021
	*GmSYP31A*	*G. max*	*A. tumefaciens*	Enhance resistance to soybean cyst nematodes	Williams82	Wang et al., 2021b
	*Hg-rps23*, *Hg-snb1*, and *Hg-cpn1*	*H. glycines*	*A. tumefaciens*	Enhance resistance to soybean cyst nematodes	Williams82	Zhang et al., 2022c
Insect resistance	*BtCry8*-like gene	*B. thuringiensis*	*A. tumefaciens*	Result in resistance to *Holotrichia parallela*	Jinong28	Qin et al., 2019
	*BtCry1Ia5*	*B. thuringiensis*	*A. tumefaciens*	Result in resistance to *Spodoptera littoralis*	Giza21 and Giza111	Moghaieb et al., 2019
Virus resistance	The coat protein gene of *MYMIV*	*Mung bean yellow mosaic India virus* (MYMIV)	*A. tumefaciens*	Result in resistance to yellow mosaic viruses	JS335	Kumari et al., 2018
	SMV P3 cistron fragment (2,529–2,830 nt)	SMV SC3	*A. tumefaciens*	Enhance resistance to multiple Potyvirus strains and isolates	Shennong9 and Williams82	Yang X. et al., 2018
	*GmeIF4E*	*G. max*	*A. tumefaciens*	Result in resistance to multiple potyvirids	Tianlong1	Gao et al., 2020
	The *AC2 gene*	*MYMIV*	*A. tumefaciens*	Enhance MYMIV resistance	JS335	Ramesh et al., 2019
	*GmKR3*	*G. max*	*A. tumefaciens*	Result in resistance to multiple viruses	Jack	Xun et al., 2019
	The protein kinase PBS1	TuMV	*A. tumefaciens*	Enhance potyvirus resistance	Williams82	Pottinger et al., 2020
	*GmVma12*	*G. max*	*A. tumefaciens*	Enhance SMV resistance	Tianlong1	Luan et al., 2020
	*GmST1*	*G. max*	*A. tumefaciens*	Enhance resistance to soybean mosaic virus strains G2 and G3	Dongnong93−046	Zhao et al., 2021
	*GmNF-YC4-2*	*G. max*	*A. tumefaciens*	Result in broad disease resistance for bacterial, viral, and fungal infections	Williams82	O’Conner et al., 2021
Fungal disease resistance	*hrpZm*	*P. syringae*	*A. tumefaciens*	Enhance tolerance to Phytophthora root and stem rot caused by *P. sojae*	Williams82 and Shennong9	Du et al., 2018
	*AtPSS1*	*A. thaliana*	*A. tumefaciens*	Result inresistance to *F. virguliforme*	Williams82	Wang et al., 2018a
	*GmCHI1A*	*G. max*	*A. rhizogenes*	Result in resistance to *P. sojae*	Williams82 (carrying Rps 1k)	Zhou et al., 2018
	*GmPI4L*	*G. max*	*A. tumefaciens*	Result in resistance to *P. sojae*	Dongnong50	Chen et al., 2019c
	*Hrf2*	*X. oryzaepv. oryzicola*	*A. tumefaciens*	Result in resistance to *P. sojae*	Shennong9	Niu et al., 2019
	*GmSnRK1.1*	*G. max*	*A. tumefaciens*	Result in resistance to *P. sojae*	Suinong10	Wang et al., 2019a
	*GmC4H1*	*G. max*	*A. rhizogenes*	Result in resistance to *P. sojae*	Conrad	Yan et al., 2019
	*TaOXO*	*T. aestivum*	*A. tumefaciens*	Enhance resistance to sclerotinia stem rot	Willams82	Yang X. et al., 2019
	*GmMYB29A2*	*G. max*	*A. rhizogenes*	Result in resistance to *P. sojae*	Harosoy, H63, Williams, and W82	Jahan et al., 2020
	*NmDef02*	*N. megalosiphon*	Biolistic method	Enhance resistance to soybean rust and anthracnose	DT-84	Soto et al., 2020
	*CmCH1*	*C. minitans*	*A. tumefaciens*	Enhanced resistance to *Sclerotiniasclerotiorum*	Williams82	Yang et al., 2020c
	*GmDR1*	*G. max*	*A. tumefaciens*	Result in broad spectrum immunity against fungal disease	Williams82	Ngaki et al., 2021
	*AtFOLT1*	*A. thaliana*	*A. tumefaciens*	Enhance resistance to broad-spectrum disease	Williams82	Kambakam et al., 2021
	*NLR* gene	*G. max*	*O. haywardense*	Result in broad-spectrum resistance to *P. sojae*	93Y21	Wang et al., 2021c
	*GmTNL16*	*G. max*	*A. rhizogenes*	Enhance resistance to *P. sojae*	Williams	Li et al., 2022
	*GmNAC1*	*G. max*	*A. tumefaciens*	Enhance resistance to *P. sojae*	Tianlong1 and Suinong10	Yu et al., 2022
Herbicide tolerance	*G10-EPSPS*	*D. radiodurans*	*A. tumefaciens*	Result in glyphosate tolerance	Zhongdou32	Xiao et al., 2019
	*G2-EPSPS* and *G10-EPSPS*	*P. fluorescens* G2	*A. tumefaciens*	Result in glyphosate tolerance	Jack	Guo et al., 2020a
	Cytochrome P450 gene*P450-N-Z1*	*C. dactylon*	*A. tumefaciens*	Result in multiple herbicides tolerance	Tianlong1	Zheng et al., 2022
Drought tolerance	*PgTIP1*	*P. ginseng*	*A. tumefaciens*	Enhance both salt and drought tolerance	Hybrid strain 4076	An et al., 2018
	*GmPIP2;9*	*G. max*	*A. tumefaciens*	Increase drought tolerance	Williams82	Lu et al., 2018
	*AtABF3*	*A. thaliana*	*A. tumefaciens*	Enhance drought tolerance	Kwangankong	Kim et al., 2018
	*GmWRKY12*	*G. max*	*A. rhizogenes*	Increase drought and salt tolerance	Williams82	Shi et al., 2018
	*GmBIN2*	*G. max*	*A. rhizogenes*	Enhance tolerance to salt and drought	Dongnong50	Wang et al., 2018b
	*GmBiP*	*G. max*	*A. tumefaciens*	Enhance drought tolerance	Conquista	Coutinho et al., 2019
	*AtYUCCA6*	*A. thaliana*	*A. tumefaciens*	Enhance drought tolerance	Kwangankong	Park et al., 2019
	*GmWRKY54*	*G. max*	*A. rhizogenes*	Enhance drought tolerance	Williams82	Wei W. et al., 2019
	*FvC5SD*	*F. velutipes*	*A. tumefaciens*	Enhance drought stress tolerance	Shennong9	Zhang et al., 2019b
	*GmNFYA5*	*G. max*	*A. rhizogenes*	Enhance drought tolerance	Williams82	Ma et al., 2020
	*AtNCED3*	*A. thaliana*	*A. tumefaciens*	Enhance drought tolerance	BRS184	Molinari et al., 2020
	*GmDREB2*	*G. max*	*A. tumefaciens*	Enhance drought tolerance	DT84	Pham et al., 2020
	*At*Δ*Kinase*	*A. thaliana*	*A. tumefaciens*	Increase tolerance to water deficit stress	Williams82	Shanmugam et al., 2020
	*HaHB4*	*H. annuus*	*A. tumefaciens*	Enhance drought tolerance with yield reduced	Williams82	Ribichich et al., 2020
	*GmNAC8*	*G. max*	*A. tumefaciens*	Enhance drought tolerance	Tianlong1	Yang et al., 2020a
	*GmbZIP2*	*G. max*	*A. tumefaciens*	Enhance tolerance to salt, drought, or cold condition	Williams82	Yang et al., 2020d
	*GmbZIP15*	*G. max*	*A. tumefaciens*	Decrease tolerance to drought and salt tolerance	C03-3	Zhang M. et al., 2020
	*Gmgma-miR398c*	*G. max*	*A. rhizogenes*	Negatively regulate drought tolerance	Williams82	Zhou et al., 2020
	*GmNTF2B-1*	*G. max*	*A. rhizogenes*	Enhance drought tolerance	Williams82	Chen et al., 2021a
	*GmMYB14*	*G. max*	*A. tumefaciens*	Enhance high-density yield and drought tolerance	Tianlong1	Chen et al., 2021b
	*GmTGA15*	*G. max*	*A. rhizogenes*	Enhance drought tolerance	Williams82	Chen et al., 2021c
	*GmPI-PLC7*	*G. max*	*A. rhizogenes*	Increase drought and salt tolerance	Williams82	Chen et al., 2021d
	*GmCIPK2*	*G. max*	*A. tumefaciens*	Enhance drought tolerance	Williams82	Xu et al., 2021
	*GsPOD40*	*G. max*	*A. tumefaciens*	Enhance drought tolerance	PI342618B/DTP and Tianlong1	Aleem et al., 2022
	*GmDREB1*	*G. max*	*A. tumefaciens*	Enhance drought tolerance	P3	Chen et al., 2022
	*sHSP26*	*G. max*	*A. tumefaciens*	Enhance drought tolerance	Jinong18	Liu et al., 2022b
	*GmDREB2*	*G. max*	*A. tumefaciens*	Enhance drought tolerance	BRS283	Marinho et al., 2022
	*GmEF8*	*G. max*	*A. rhizogenes*	Enhance drought and heat tolerance	Williams82	Zhang et al., 2022a
Salt and other stress tolerance	*PgTIP1*	*P. ginseng*	*A. tumefaciens*	Enhance both salt and drought tolerance	Hybrid strain 4076	An et al., 2018
	*ZmGB1*	*Z. mays*	*A. tumefaciens*	Enhance tolerance to abiotic stress	**\**	Castiglioni et al., 2018
	*GmWRKY12*	*G. max*	*A. rhizogenes*	Increase drought and salt tolerance	Williams82	Shi et al., 2018
	*AtXTH31*	*A. thaliana*	*A. tumefaciens*	Enhance tolerance to flooding stress	Maverick	Song et al., 2018
	*GmBIN2*	*G. max*	*A. rhizogenes*	Enhance tolerance to salt and drought	Dongnong50	Wang et al., 2018b
	*MsWRKY11*	*M. sativa* (alfalfa)	*A. tumefaciens*	Enhance salt tolerance	Dongnong50	Wang et al., 2018c
	*GmHsp90A2*	*G. max*	*A. tumefaciens*	Increase tolerance to heat stress	Qihuang22	Huang et al., 2019
	*AtAVP1* and *AtNHX1*	*A. thaliana*	*A. tumefaciens*	Increase salt tolerance	DT26	Nguyen N. T. et al., 2019
	*GmDREB-6*	*G. max*	*A. tumefaciens*	Enhance salt tolerance	DT84	Nguyen Q. H. et al., 2019
	*GsCLC-c2*	*G. soja*	*A. tumefaciens*	Enhance salt tolerance	N23674	Wei P. et al., 2019
	*GmERF135*	*G. max*	*A. rhizogenes*	Enhance salt tolerance	Tiefeng8	Zhao et al., 2019a
	*GmCDF1*	*G. max*	*A. rhizogenes*	Negatively regulate salt tolerance	Kefeng1 and Nannong1138–2	Zhang et al., 2019c
	*GmSAP16*	*G. max*	*A. tumefaciens*	Enhance drought and salt tolerance	Williams82	Zhang et al., 2019d
	*J*	*G. max*	*A. tumefaciens*	Increase salt tolerance	Huaxia3	Cheng et al., 2020
	*GsSnRK1*	*G. soja*	*A. tumefaciens*	Increase salt and alkaline stresses tolerance	Dongnong50	Feng et al., 2020
	*GmMYB68*	*G. max*	*A. tumefaciens*	Increase salt and alkaline stresses tolerance	Williams82	He et al., 2020
	*Gs5PTase8*	*G. soja*	*A. rhizogenes*	Enhance salt tolerance	Mengjin1 and Union	Jia et al., 2020
	*GsAAE3*	*G. soja*	*A. rhizogenes*	Increase tolerance to Cd and Al stresses	BW69	Xian et al., 2020
	*GmbZIP2*	*G. max*	*A. tumefaciens*	Enhance tolerance to salt, drought, or cold condition	Williams82	Yang et al., 2020d
	*GsJAZ2*	*G. soja* (G07256)	Biolistic method	Enhance tolerance to alkaline stress	HF55	Zhao et al., 2020
	*GmbZIP15*	*G. max*	*A. tumefaciens*	Decrease tolerance to drought and salt tolerance	C03-3	Zhang M. et al., 2020
	*GmPI-PLC7*	*G. max*	*A. rhizogenes*	Increase drought and salt tolerance	Williams82	Chen et al., 2021d
	*AgGlpF*	*A. glaucus*	*A. tumefaciens*	Enhance salt tolerance	Williams82	Li et al., 2021a
	*GmNAC06*	*G. max*	*A. rhizogenes*	Enhance salt tolerance	Williams82	Li et al., 2021b
	*GsCLC-c2*	*G. soja*	*A. tumefaciens*	Enhance Cl^–^/salt tolerance	BB52	Liu et al., 2021
	*GsBET11a*	*G. soja*	*A. tumefaciens*	Enhance salt tolerance	G07256 and Dongnong50	Sun X. et al., 2021
	*GmNHX5*	*G. max*	*A. rhizogenes*	Enhance salt tolerance	Jidou-7	Sun T. et al., 2021
	*GmAKT1*	*G. max*	*A. rhizogenes*	Enhance salt tolerance	Dongnong50	Wang et al., 2021e
	*GmbHLH3*	*G. max*	*A. rhizogenes*	Enhance Cl^–^/salt tolerance	N23674	Liu et al., 2022c
	*GmEF8*	*G. max*	*A. rhizogenes*	Enhance drought and heat tolerance	Williams82	Zhang et al., 2022a

*\ means not available.*

## Challenges and Future Directions in Soybean Transformation

Although much effort has been made to improve the transformation systems for soybean, there are some challenges such as genotype flexibility, low transformation frequency, time to time chimerism in T_0_ transgenic plants, and availability of a system for new breeding technologies such as genome editing.

### Genotype Flexibility

Like in other recalcitrant plant species, genotype inflexibility has been an obstacle that restricted the scope of soybean transformation. The ideal soybean transformation target material for trait improvement would be any elite variety with excellent agronomic characteristics. However, most reliable transformations are still based on specific genotypes although genotypes amenable to transformation have expanded to some preferred genotypes. For example, in the early stage, successful *Agrobacterium*-mediated transformation occurred in several genotypes and their derivatives such as Williams, Williams79, and Williams82 (Paz et al., 2004, 2006). High-efficiency *Agrobacterium*-mediated transformation is only achieved in a limited number of elite lines (Zhang et al., 2014; Arun et al., 2015, 2016; Yang J. et al., 2016; Li et al., 2017; Chen et al., 2018b). High-efficiency genotypes possess greater susceptibility to *Agrobacterium* infection, which has been confirmed in many reports (Jia et al., 2015; Yang J. et al., 2016; Yang X. F. et al., 2016; Zhao et al., 2019b). The competency of cotyledons of seeds to *Agrobacterium* infection and the ability to regenerate plants are key factors. These may be determined by cell defense response, including attachment of *A. tumefaciens* to plant cells, plant signals sensed by *A. tumefaciens*, regulating *vir* gene expression, T-DNA/virulence protein transport or initial contact of *A. tumefaciens* to plants and cytoplasmic trafficking, and nuclear import of T-DNA and effector proteins (Hwang et al., 2017). An important step to enhance the transformation efficiency of recalcitrant genotypes is to improve the genotypes’ susceptibility to *Agrobacterium* infection. Many commonly used treatments to increase transformation efficiency such as heat shock, cold shock, antioxidants, and hypoxia may act by suppression of cellular response to *Agrobacterium* infection (Zhang et al., 2013). Combinations of various positive factors discovered or developed recently have promoted *Agrobacterium*-mediated soybean transformation to extend genotype scope (Table 1). For example, transgenic events have been obtained from 19 out of 20 genotypes based on an improved protocol (Pareddy et al., 2020) and 7 out of 8 genotypes (Zhao et al., 2019b). One important progress in these reports is that over 5% of TFs were obtained in nearly half of these genotypes. The second factor that affects genotype flexibility is the regeneration ability of donor genotypes, which restricts TFs for both *Agrobacterium*-mediated and biolistic transformations. Increasing the amenability of many soybean genotypes to regenerate may be conducted by either adding some specific chemicals in the culture medium described above or using plant regeneration factors or regeneration booster genes. Significant progress has been made to improve transformations from various tissue types using plant regeneration factors such as maize (*Zea mays*) morphogenic genes, *Baby boom* (BBM) and *Wuschel2* (*WUS2*) genes in maize plant (Lowe et al., 2016), and plant growth regulators such as *GROWTH-REGULATING FACTORS* (*GRF*) genes used in monocot and dicot species including soybean (Gordon-Kamm et al., 2019; Debernardi et al., 2020; Kong et al., 2020). Use of these genes significantly increased transformation frequency and reduced genotype obstacle for transformation, providing a good solution for genotype-inflexibility bottleneck in transformation of crops including soybean. For example, introducing *AtGRF5* and *GRF5* orthologs into soybean cells could improve regeneration and, hence, increase transformation TFs significantly (Kong et al., 2020). GRFs can also enhance shoot organogenesis and callus regeneration, which has been confirmed in dicots including sugar beet, canola, and sunflower. Meanwhile, somatic embryogenesis can be promoted using some genes introduced into explants in soybean, such as soybean orthologs of the *Arabidopsis* (*A. thaliana*) MADS box genes *AGAMOUS-Like15* (*GmAGL15*) and *GmAGL18*, which can also expand soybean genotypes suitable for transformation, especially for biolistic transformation (Zheng and Perry, 2014). Transformation bypass tissue culture such as *in planta* transformation is an alternative way to overcome genotype inflexibility in soybean (Liu et al., 2009; Mangena, 2019). Nanotechnology-based transformation can also be employed to overcome host range limitation including genotype inflexibility, and can simplify delivery way using pollen channel, and highly increase efficiency (Wang and Zhao, 2019). By integration of multiple-omics technologies, genes related to transformation efficiency should be discovered for increasing transformation efficiency. Use of the novel bacterium *O. haywardense* H1 may also increase the genotype scope for transformation, since it was claimed to be less genotype sensitive when it was used for soybean transformation (Cho et al., 2022).

### Low Transformation Frequency

The average TF for varieties (genotypes) reported is lower than 5%, although improvements have been made by modifying the main factors described above (Table 1). Since the biolistic method tends to use an embryogenic callus as explant because of less chimerism compare to an embryo axis, TFs for biolistic transformation are dependent on the success of embryogenic callus induction for a specific genotype. Therefore, the main focus to improve TFs is to select genotypes that are amenable to embryogenic callus induction, or to stimulate a genotype to produce an embryogenic callus. As described above, the regeneration booster provides a new way to induce an embryogenic callus without genotype limitation, which has been confirmed in monocot plants (Lowe et al., 2018; Gordon-Kamm et al., 2019; Debernardi et al., 2020). Enhancement of TFs for soybean Agrobacterium-mediated transformation is mainly achieved by improving regeneration rates of explants and increasing the susceptibility of explants to *Agrobacterium*. Half-seed explants have been the major choice, because these explants could provide more nutrition and less damage than cotyledonary nodes (Table 1). Continuously modifying MS-based culture medium composition (Murashige and Skoog, 1962), especially by addition of chemicals discovered through the study of omics, has played a big role in TF improvement, and has been summarized in the section above. Combinations of many factors have promoted the TFs of soybean transformation (Table 1). More efforts should be made to increase the average TFs close to that of other major crops. Again, the morphogenic genes including GRFs described above may play an important role in enhancing soybean transformation frequency.

### Chimerism in T_0_ Transgenic Plant

Chimerism in legume transformation is fairly common, which causes non-transmission of transgenes to subsequent generations either completely or at a lower ratio expected by Mendelian genetics. Therefore, minimizing chimerism in transgenic plants is required to obtain transmission of transgenes to the T_1_ generation. In soybean, *Agrobacterium*-mediated transformation of cotyledonary nodes by organogenesis has been extensively conducted for transgenic production in research and commercial product development (Barwale et al., 1986; Homrich et al., 2012; Yamada et al., 2012; Lee et al., 2013; Mariashibu et al., 2013; Mangena et al., 2017). Plant regeneration by organogenesis with an explant containing an embryo axis may be the main cause, since shoots regenerated from soybean shoot tips were derived from 3 superimposed cellular layers (L1, L2, and L3) in the original apical meristem (Christou, 1990; Christou and Mccabe, 1992). Transformed cells existed primarily in the L1 and L2 layers but not in the L3 layer of the apical meristems of regenerated shoots, indicating possible escape in the regenerated shoots during transformation, and this chimerism has been confirmed (Mccabe et al., 1988; Sato et al., 1993). Currently, the chimerism in transgenic soybean is still a major concern in the research community, and inheritance study has been always an important part in transformation protocol development (Pareddy et al., 2020). Improvement for reducing escapes or chimeric rate has been made when strict select stringency was used, especially some new selectable markers/reagents such as AHAS/imazapyr (Aragão et al., 2000; Rech et al., 2008), EPSPS/glyphosate (Martinell et al., 1999; Guo et al., 2015, 2020a; Soto et al., 2017), and AADA/spectinomycin (Martinell et al., 2002). Meanwhile, the modified protocols made use of specific explants, such as somatic embryogenic calli, to reduce the chance of infection with cells at the late development stage, and combined proper selection of chemical agents with high stringency to decrease escape rate dramatically, which led to more than 90% T_0_ transgenic plants transmitting their transgenes into T_1_ generation (Soto et al., 2017; Chennareddy et al., 2018; Guo et al., 2020a). Therefore, transgenic soybean with chimeric issues may due to insufficient selection that existed in various protocols.

### Development of Transformation Method for New Breeding Technology

Genome editing is the recent advancement in genome engineering, which has revolutionized crop research and plant breeding. GE, through site-specific nucleases (SSNs), can precisely make changes in targeted genome sequence sites by disruption including insertion and deletion, base changes, sequence replacement, and sequence insertion. SSNs include zinc-finger nucleases (ZFNs), transcription activator-like effector nucleases (TALENs), and CRISPR/CAS. GE is a fast-developing technology that will potentially play an important role in genomics study and will create opportunities for rapid development of elite cultivars with desired traits. The development of soybean GE has been reviewed in Xu et al. (2020). Recent GE applications in soybean for trait improvement have been summarized in Table 3. For example, function analysis of photo period-related genes such as *LHY* homologs, *J* and *E1*, and *tof 16* (*Time of Flowering 16*) using GE technology showed that more than 80% accessions in low latitude harbor the mutations of *tof16* and *J*, which suggests that loss of functions of *Tof16* and *J* is the major genetic basis of soybean adaptation into tropics. Therefore, maturity and yield traits can be quantitatively improved by modulating the genetic complexity of various alleles of *LHY* homologs, *J*, and *E1* (Bu et al., 2021; Dong et al., 2021). The findings uncover the adaptation trajectory of soybean from its temperate origin to the tropics. Knockout of *GmJAG1*, which controls the number of seeds per pod (NSPP), increases by over 8% the yield of a Chinese variety, Huachun 6 (Cai et al., 2021). *GmMs1* KO events in soybean were created, which showed male sterility phenotype (Jiang et al., 2021; Nadeem et al., 2021). SCN-resistant mechanisms such as t-SNAREs binding Rhg1 α-SNAP (Dong et al., 2020) and WI12_*Rhg*1_ interacting with DELLAs (Dong and Hudson, 2022) were found using GE as a tool. Targeted chromosome cleavage by CRISPR/Cas9 can conceivably induce rearrangements and, thus, emergence of new resistance gene paralogs. CRISPR/Cas9-mediated chromosome rearrangements in nucleotide-binding-site-leucine-rich-repeat (NBS-LRR) gene families of soybean produced a new disease-resistant gene (Nagy et al., 2021). Raffinose family oligosaccharides (RFOs) are major soluble carbohydrates in soybean seeds that cannot be digested by humans and other monogastric animals. Double mutation events, knockouts in two soybean galactinol synthase (GOLS) genes, *GmGOLS1A* and its homeolog *GmGOLS1B*, showed a reduction in the total RFO content of soybean seeds from 64.7 to 41.95 mg/g dry weight, a 35.2% decrease (Le et al., 2020). This product improved the soybean nutrition quality. Two transcription systems were also tested in soybean including the single transcriptional unit (STU), SpCas9 and sgRNA are driven by only one promoter, and in the conventional system, the two-component transcriptional unit (TCTU), SpCas9, is under the control of a pol II promoter, and sgRNAs are under the control of a pol III promoter. The results showed that the STU is more efficient (Carrijo et al., 2021). Cpf1 (Cas12a) systems have also been established in soybean for GE (Duan et al., 2021; Kim and Choi, 2021). Meanwhile, different GE systems for soybean have been established using specific editing reagent delivery methods developed for soybean transformation, which produce transgene-free GE events either with the biolistic method (Adachi et al., 2021) and selectable marker-free GT systems by *O. haywardense* H1-8-mediated delivery (Kumar et al., 2022), or by organ-specific editing using an egg cell-specific promoter (Zheng et al., 2020). All these GE studies on soybean demonstrate that the ability to conduct genome editing directly depends on plant transformation technologies, since recovery of stable events with the target gene edited is normally based on available transformation systems including editing reagent delivery and edited event regeneration. GE has the potential to avoid many regulatory issues regarding transgenics if specific editing reagents are used. Based on the CRISPR/CAS system, gRNA in the form of *in vitro*-synthesized RNA molecule, together with Cas9 as DNA construct, can be stably integrated into the host genome and constitutively expressed, which might lead to a transgenic event for a GE event. This issue can be resolved by introduction of editing tools without genomic integration or transient expression. Transgene-free or DNA-free edited events in many crops can now be obtained either by delivering the RNA form of sgRNA and Cas9 or Cas9 protein (RNP) using the biolistic method, or by protoplast transfection (Chen et al., 2019a; Xu et al., 2020; Gao, 2021; Kim and Choi, 2021). Transgene-free events can also be recovered with the *Agrobacterium*-mediated method without selection (Liang et al., 2017). However, genotype flexibility limitation is a major issue for soybean GE in the biolistic method, and low TF for some elite varieties is the main hurdle in the *Agrobacterium*-mediated method.

**TABLE 3:** List of soybean genes edited for functional genetics study and trait improvement using genome editing technology.

Trait	Gene/targeting location	GE platform	Delivery method	Edited events	Editing outcomes	References
**Yield**						
Plant architecture	*GmLHY1a* (*Glyma.16G017400*), *GmLHY1b* (*Glyma.07G048500*), *GmLHY2a* (*Glyma.19G260900*), and *GmLHY2b* (*Glyma.03G261800*)	CRISPR/Cas9	*A. tumefaciens*	Whole plant	Knockout (multiplex)	Cheng et al., 2019
	*GmSPL9a* (*Glyma.02G177500*), *GmSPL9b* (*Glyma.09G113800*), *GmSPL9c* (*Glyma.03G143100*), and *GmSPL9d* (*Glyma.19G146000*)	CRISPR/Cas9	*A. tumefaciens*	Whole plant	Knockout (multiplex)	Bao et al., 2019
	*GmAP1a* (*Glyma.16G091300*), *GmAP1b* (*Glyma.08G269800*), *GmAP1c* (*Glyma.01G064200*), and *GmAP1d* (*Glyma.02G121600*)	CRISPR/Cas9	*A. tumefaciens*	Whole plant	Knockout (multiplex)	Chen et al., 2020d
Seed weight and organ size	*GmPPD1* (*Glyma.10G244400*) and *GmPPD2* (*Glyma.20G150000*)	CRISPR/Cas9	*A. tumefaciens*	Whole plant	Knockout (multiplex)	Kanazashi et al., 2018
	*GmSWEET10a* (*Glyma.15G049200*) and *GmSWEET10b* (*Glyma.08G183500*)	CRISPR/Cas9	*A. tumefaciens*	Whole plant	Knockout (multiplex)	Wang et al., 2020c
	*GmKIX8-1* (*Glyma.17G112800*)	CRISPR/Cas9	*A. tumefaciens*	Whole plant	Knockout	Nguyen et al., 2021
Seed number	*GmJAG1* (*Glyma.20G25000*) and *GmJAG2* (*Glyma.10G42020*)	CRISPR/Cas9	*A. tumefaciens*	Whole plant	Knockout (multiplex)	Cai et al., 2021
Photoperiod	*GmFT2a* (*Glyma.16G26660*)	CRISPR/Cas9	*A. tumefaciens*	Whole plant	Knockout	Cai et al., 2018
	*GmE1* (*Glyma.06G207800*)	CRISPR/Cas9	*A. tumefaciens*	Whole plant	Knockout	Han et al., 2019
	*GmFT2a* (*Glyma.16G26660*) and *GmFT5a* (*Glyma.16G04830*)	CRISPR/Cas9	*A. tumefaciens*	Whole plant	Knockout (multiplex)	Cai et al., 2020b
	*GmFT2a* (*Glyma.16G150700*) and *GmFT4* (*Glyma.08G363100*)	BE base editor	*A. tumefaciens*	Whole plant	Base editing	Cai et al., 2020a
	*GmFT2b* (*Glyma.16G26690*)	CRISPR/Cas9	*A. tumefaciens*	Whole plant	Knockout	Chen et al., 2020c
	*GmAP1a* (*Glyma.16G091300*), *GmAP1b* (*Glyma.08G269800*), *GmAP1c* (*Glyma.01G064200*), and *GmAP1d* (*Glyma.02G121600*)	CRISPR/Cas9	*A. tumefaciens*	Whole plant	Knockout (multiplex)	Chen et al., 2020d
	*GmPRR37* (*Glyma.12G073900*)	CRISPR/Cas9	*A. tumefaciens*	Whole plant	Knockout	Wang et al., 2020b
	*GmLUX1* (*Glyma.12G060200*) and *GmLUX2* (*Glyma.11G136600*)	CRISPR/Cas9	*A. tumefaciens*	Whole plant	Knockout (multiplex)	Bu et al., 2021
	*GmLNK2a* (*Glyma.04G141400*), *GmLNK2b* (*Glyma.11G154700*), *GmLNK2c* (*Glyma.13G199300*), and *GmLNK2d* (*Glyma.15G237600*)	CRISPR/Cas9	*A. tumefaciens*	Whole plant	Knockout (multiplex)	Li et al., 2021c
**Nutrition and quality**						
Storage protein	*Glyma.20G148400*, *Glyma.20G146200*, *Glyma.10G246300*, *Glyma.20G148200*, *Glyma.10G037100*, *Glyma.03G163500*, *Glyma.19G164900*, *Glyma.13G123500*, and *Glyma.19G164800*	CRISPR/Cas9	*A. rhizogenes*	Hairy root	Knockout (multiplex)	Li et al., 2019
Seed oil	*GmFAD2-1A* (*Glyma.10G278000*) and *GmFAD2-1B* (*Glyma.20G111000*)	TALENs	*A. rhizogenes* and *disarmed A. rhizogenes*	Hairy root and whole plant	Knockout (multiplex)	Haun et al., 2014
	*GmFAD2-2*	CRISPR/Cas9	*A. tumefaciens*	Whole plant	Knockout	al Amin et al., 2019
	*GmFAD2-1A* (*Glyma.10G278000*)	ZFNs	Biolistic method	Whole plant	Knock in (NHEJ)	Bonawitz et al., 2019
	*GmFAD2-1A* (*Glyma.10G278000*) and *GmFAD2-1B* (*Glyma.20G111000*)	CRISPR/Cas9	*A. tumefaciens*	Whole plant	Knockout (multiplex)	Do et al., 2019
	*GmGOLS1A* (*Glyma.03G222000*) and *GmGOLS1B* (*Glyma.19G219100*)	CRISPR/Cas9	*A. tumefaciens*	Whole plant	Knockout (multiplex)	Le et al., 2020
	*GmFAD2–1A* (*Glyma.10G278000*) and *GmFAD2–2A* (*Glyma.19G147300*)	CRISPR/Cas9	*A. tumefaciens*	Whole plant	Knockout (multiplex)	Wu et al., 2020
	*GmFATB1a* (*Glyma.05G012300*) and *GmFATB1b* (*Glyma.17G012400*)	CRISPR/Cas9	*A. tumefaciens*	Whole plant	Knockout (multiplex)	Ma J. et al., 2021
	*Glyma.15G117700*	CRISPR/Cas9	*A. tumefaciens*	Whole plant	Knockout	Qu et al., 2021
Bean flavor-free soybean	*GmLox1* (*Glyma.13G347600*), *GmLox2* (*Glyma.13G347500*), and *GmLox3* (*Glyma.15G026300*)	CRISPR/Cas9	*A. tumefaciens*	Whole plant	Knockout (multiplex)	Wang et al., 2020a
**Disease resistance**						
Cyst nematode resistance	*GmSyn12* (*Glyma.12G194800*), *GmSyn13* (*Glyma.13G307600*), *GmSyn16* (*Glyma.16G154200*), and *GmSyn02* (*Glyma.02G072900*)	CRISPR/Cas9	*A. rhizogenes*	Hairy root	Knockout (multiplex)	Dong et al., 2020
	*Rps1 families* (*Glyma.03G034400*, *Glyma.03G0034800*, *Glyma.03G039200*, *Glyma.03G039500*, *Glyma.03G037100*, *Glyma.03G037300*, *Glyma.03G037400*, *Glyma.03G037400*, *Glyma.03G037000*, *Glyma.03G034500*, *Glyma.03G039300*, *Glyma.03G045700*, *Glyma.03G043600*, *Glyma.03G045300*, *Glyma.03G043000*, *Glyma.03G043500*, *Glyma.03G044000*, *Glyma.03G043200*, *Glyma.03G045000*, *Glyma.03G046500*, *Glyma.03G047000*, *Glyma.03G043900*) and *Rpp1L families* (*Glyma.18G281700*, *Glyma.18G281600*, *Glyma.18G281500*, and *Glyma.18G280300*)	CRISPR/Cas9	*A. tumefaciens*	Whole plant	Knockout (multiplex)	Nagy et al., 2021
	*Rhg1*-locus (*Glyma.18G02270*), *DELLA18* (*Glyma.18G040000*), and *DELLA11* (*Glyma.11G216500*)	CRISPR/Cas9	*A. rhizogenes*	Hairy root	Knockout (multiplex)	Dong and Hudson, 2022
Insect resistance	*GmUGT* (*Glyma.07G110300*)	CRISPR/Cas9	*A. tumefaciens*	Whole plant	Knockout	Zhang et al., 2022b
**Abiotic stress tolerance**						
Drought tolerance	*GmLHY1a* (*Glyma.16G017400*), *GmLHY1b* (*Glyma.07G048500*), *GmLHY2a* (*Glyma.19G260900*), and *GmLHY2b* (*Glyma.03G261800*)	CRISPR/Cas9	*A. tumefaciens*	Whole plant	Knockout (multiplex)	Wang et al., 2021a
Salt tolerance	*GmNAC06* (*Glyma06G21020*)	CRISPR/Cas9	*A. rhizogenes*	Hairy root	Knockout	Li et al., 2021b
**Nitrogen fixation**						
	*GmNSP1a* (*Glyma.07G039400*) and *GmNSP1b* (*Glyma.16G008200*)	CRISPR/Cas9	*A. tumefaciens*	Whole plant	Knockout (multiplex)	He et al., 2021
Herbicide resistance	*GmALS1* (*Glyma.04G37270.1*), *GmALS2* (*Glyma.06G17790.1*), *GmALS3* (*Glyma.13G31470.1*), and *GmALS4* (*Glyma.15G07860.1*)	CRISPR/Cas9	Biolistic method	Whole plant	Knockin (HDR)	Li et al., 2015
Root nodulation	*GmRIC1* (*Glyma.13G292300*), *GmRIC2* (*Glyma.06G284100*), *GmRDN1-1* (*Glyma.02G279600*), *GmRDN1-2* (*Glyma.14G035100*), and *GmRDN1-3* (*Glyma.20G040500*)	CRISPR/Cas9	*A. tumefaciens*	Whole plant	Knockout (multiplex)	Bai et al., 2020
	*GmSPL9d* (*Glyma.19G146000*) and *GmmiR156*	CRISPR/Cas9	*A. rhizogenes*	Hairy root	Knockout (multiplex)	Yun et al., 2022
Allergy reduction	*Gly m Bd 28K* (*Glyma.U020300*) and *Gly m Bd 30K* (*Glyma.08G116300*)	CRISPR/Cas9	*A. tumefaciens*	Whole plan*t*	Knockout (multiplex)	Sugano et al., 2020
**GE platform adoption in soybean**	*GmDCL1a* (*Glyma.03G42290*), *GmDCL1b* (*Glyma.19G45060*), *GmDCL4a* (*Glyma.17G11240*), *GmDCL4b* (*Glyma.13G22450*), *GmRDR6a* (*Glyma.04G07150*), *GmRDR6b* (*Glyma.06G07250*), and *GmHEN1a* (*Glyma.08G08650*)	ZFNs	*A. rhizogenes*	Hairy root	Knockout (multiplex)	Curtin et al., 2011
	*GmDCL4a* (*Glyma.17G11240*) and *GmDCL4b* (*Glyma.13G22450*)	ZFNs	*A. rhizogenes*	Hairy root	Knockout	Sander et al., 2011
	*Bar* transgene, *GmFEI1* (*Glyma.01G35390*), *GmFEI2* (*Glyma.09G34940*), and *GmSHR*	CRISPR/Cas9	*A. rhizogenes*	Hairy root	Knockout (multiplex)	Cai et al., 2015
	*GmGS* (*Glyma.18G04660* and *Glyma.18G041100*) and *GmCHI20* (*Glyma.20G38560* and *Glyma.20G241500*)	CRISPR/Cas9	*A. rhizogenes*	Hairy root	Knockout	Michno et al., 2015
	*GFP* transgene, *Glyma07g14530*, *01gDDM1* (*Glyma.11G38150*), *11gDDM1* (*Glyma.11G07220*), *Glyma04g36150*, *Glyma06g18790*, *miR1509*, and *miR1514*	CRISPR/Cas9	*A. rhizogenes*	Hairy root	Knockout	Jacobs et al., 2015
	*Glyma.06G14180*, *Glyma.08G02290*, and *Glyma.12G37050*	CRISPR/Cas9	*A. rhizogenes*	Hairy root	Knockout (multiplex)	Sun et al., 2015
	*GmPDS11* (*Glyma.11G253000*) and *GmPDS18* (*Glyma.18G003900*)	TALENs	*A. tumefaciens*	Whole plant	Knockout	Du et al., 2016
	*FAD2-1A* (*Glyma.10G42470*) and *FAD2-1B* (*Glyma.20G24530*)	CRISPR/AsCpf1 or LpCpf1	Protoplast transfection	Protoplast	Knockout (RNP)	Kim et al., 2017
	*GmIPK1* (*Glyma.14G072200*) and *GmIPK2* (*Glyma.12G240900*) (STU and TCTU system*)	CRISPR/Cas9	*A. rhizogenes*	Hairy root	Knockout (multiplex)	Carrijo et al., 2021
	*Glyma.15G249000* and *Glyma.13G259100*	CRISPR/Cas9	*A. rhizogenes*	Hairy root	Knockout (multiplex)	Luo et al., 2021
	*GmPDS11g* (*Glyma.11g253000*) and *GmPDS18g* (*Glyma.18g003900*)	CRISPR/Cas9	*A. tumefaciens*	Whole plant	Knockout (multiplex)	Lu and Tian, 2022
Transgene-free edited events	Target sites DD38 and DD51	CRISPR/Cas9	*O. haywardense H1-8*		Targeted insertion	Kumar et al., 2022
	*Gly m Bd 30K* (*Glyma.08G116300*)	CRISPR/Cas9	Biolistic method	Whole plant	Knockout	Adachi et al., 2021
Egg cell promoter driving Cas9	*GmAGO7a* (*Glyma.01G053100*) and *GmAGO7b* (*Glyma.02G111600*)	CRISPR/Cas9	*A. rhizogenes andA. tumefaciens*	Hairy root and whole plant	Knockout (multiplex)	Zheng et al., 2020
Targeted deletions of DNA fragments	*GmFT2a* (*Glyma.16G26660*) and *GmFT5a* (*Glyma.16G04830*)	CRISPR/Cas9	*A. tumefaciens*	Whole plant	Knockout (4.5 kb in *GmFT2a*)	Cai et al., 2018
Growth of soybean trichomes	*GmCPR5* (*Glyma.06G145800*)	CRISPR/Cas9	Biolistic method	Whole plant	Knockout	Campbell et al., 2019
Fertility	*GmMs1* (*Glyma.13G114200*)	CRISPR/Cas9	Biolistic method	Whole plant	Knockout	Nadeem et al., 2021
	*GmMs1* (*Glyma.13G114200*)	CRISPR/Cas9	Biolistic method	Whole plant	Knockout	Jiang et al., 2021
miRNA pathway and small RNA processing	*GmDCL1a* (*Glyma.03G42290*), *GmDCL1b* (*Glyma.19G45060*), *GmDCL4a* (*Glyma.17G11240*), *GmDCL4b* (*Glyma.13G22450*), *GmRDR6a* (*Glyma.04G07150*), *GmRDR6b* (*Glyma.06G07250*), *GmHEN1a* (*Glyma.08G08650*), and *GFP* transgene	ZFNs	*A. rhizogenes*	Hairy root	Knockout	Curtin et al., 2011
	*GmDRB2a* (*Glyma.12G075700*), *GmDRB2b* (*Glyma.11G145900*), *GmDCL3a* (*Glyma.04G057400*), *GmHEN1a* (*Glyma.08G081600*), and *GmHEN1b* (*Glyma.05G126600*)	CRISPR/Cas9	*A. rhizogenes*	Hairy root	Knockout (multiplex)	Curtin et al., 2018
	*GmDCL2a* (*Glyma.09G025400*), *GmDCL2b* (*Glyma.09G025300*), and *GmDCL3a* (*Glyma.04G057400*)	TALENs	Disarmed *A. rhizogenes*	Whole plant	Knockout (multiplex)	Curtin et al., 2018
Sucrose export related embryo development	*GmSWEET15a* (*Glyma.05G126600*) and *GmSWEET15b* (*Glyma.05G1266000*)	CRISPR/Cas9	*A. tumefaciens*	Whole plant	Knockout (multiplex)	Wang et al., 2019b
Circadian rhythmicity	*GmLCLa1* (*Glyma.16G01980*), *GmLCLa2* (*Glyma.07G05410*), *GmLCLb1* (*Glyma.03G42260*), and *GmLCLb2* (*Glyma.19G45030*)	CRISPR/Cas9	*A. tumefaciens*	Whole plant	Knockout (multiplex)	Wang et al., 2020e
Soybean knockout library	70 sgRNAs to target 102 genes	CRISPR/Cas9	*A. tumefaciens* (pooled)	Whole plant	Knockout (multiplex)	Bai et al., 2020

*STU, single transcriptional unit; SpCas9 and sgRNA are driven by only one promoter; and the two-component transcriptional unit (TCTU) in the conventional system, and SpCas9 and sgRNA are under the control of different promoters.

## Conclusion

As summarized above, development of soybean transformation protocols, which pose genotype-flexibility and relatively high efficiency and can easily be adapted in any laboratory, is still a main task for researchers. Reducing biological restrictions such as genotype dependence or tissue-specific and method restrictions will eventually lead to transformation automation and versatile and high throughput, which will facilitate the application of next-generation breeding technologies such as genome editing for soybean improvement. These goals may be achieved with fast progress in fundamental research to unravel basic biological process and genetic background, especially when more regeneration regulators such as morphogenic genes are identified. Transgenic soybean in which various genes can be manipulated will accelerate the validation of gene function in the context of complex gene networks at different plant developmental stages, which will accelerate the understanding of the mechanism of soybean cell regeneration, and it is beneficial for us to modify transformation protocols. New technologies like nanoparticle delivery also bring us hope to break through these barriers as well as the transformation bypass method.

## Author Contributions

HX and YG collected the materials. HX drew the figures and tables. YR wrote the manuscript. LQ and YR designed the article structure. All authors contributed to the article and approved the submitted version.

## Conflict of Interest

HX and YR were employed by Tianjin Genovo Biotechnology Co., Ltd. The remaining authors declare that the research was conducted in the absence of any commercial or financial relationships that could be construed as a potential conflict of interest.

## Publisher’s Note

All claims expressed in this article are solely those of the authors and do not necessarily represent those of their affiliated organizations, or those of the publisher, the editors and the reviewers. Any product that may be evaluated in this article, or claim that may be made by its manufacturer, is not guaranteed or endorsed by the publisher.

## Footnotes

1
https://www.sourcewatch.org/index.php?title=SourceWatch
